# Structural analysis of uridine modifications in solved RNA structures

**DOI:** 10.1093/nargab/lqaf197

**Published:** 2026-01-10

**Authors:** Sebastian J Arteaga, Brent M Znosko

**Affiliations:** Department of Chemistry, Saint Louis University, 3501 Laclede Ave., Saint Louis, MO 63103, United States; Department of Chemistry, Saint Louis University, 3501 Laclede Ave., Saint Louis, MO 63103, United States

## Abstract

Naturally occurring uridine modifications in RNA play critical roles in modulating RNA stability, translation, and immune responses. While detection methods have advanced, a comprehensive structural analysis across experimentally determined RNA 3D structures remains limited. In this study, we systematically examined six uridine modifications—pseudouridine (PSU), 5-methyluridine (5MU), 3-methyluridine (UR3), O2′-methyluridine (OMU), 4-thiouridine (4SU), and 5,6-dihydrouridine (H2U)—using data from the Research Collaboratory for Structural Bioinformatics Protein Data Bank. After curation, we identified 2982 PSU, 736 5MU, 232 UR3, 429 OMU, 314 4SU, and 171 H2U residues across RNA-containing structures. These modifications were primarily found in ribosomal and transfer RNAs, often localized within hairpin secondary structures. Sugar pucker analysis revealed modification-specific preferences for C3′-*endo* and C2′-*endo* conformations. To assess structural impacts, we generated sequence-representative structures and compared modified versus unmodified forms using all-atom root-mean-square deviation analysis. Most modifications showed high similarity to their unmodified counterparts (RMSD $ \le $ 1.0 Å), though deviations were notable for certain PSU-, 5MU-, and 4SU-containing motifs. Despite overall similarity, interaction differences were observed between modified and canonical uridines. This work provides a detailed structural overview of uridine modifications, offering insights into their conformational behavior and implications for RNA function. These findings may inform future efforts in RNA-targeted therapeutics and structural biology.

## Introduction

Native nucleic acids often contain a variety of noncanonical nucleotides, which differ from the standard bases A, C, G, U, and T. The most common nucleic acid modifications include methylation [1], deamination [2], and oxidation events [3]. In DNA, methylation is the most prevalent modification and is frequently associated with cancer [4]. In RNA, methylation, along with numerous other types of modifications [5, 6], occurs both post-transcriptionally and co-transcriptionally. Naturally occurring modifications are commonly found in transfer RNA (tRNA), ribosomal RNA (rRNA), and small nuclear RNA (snRNA), with a single tRNA molecule containing an average of 13 modifications [7, 8]. To understand the mechanisms behind these modifications and their biological implications, Bujnicki and colleagues developed the MODOMICS database [9–11], which catalogs ~170 RNA modifications, detailing their associated pathways, reactions, and links to human diseases [9, 11]. Many of these modifications are produced by enzymes that convert canonical bases and sugars to their modified, noncanonical counterparts.

Research on RNA modifications has been ongoing for over 50 years [12]. In the past three decades, significant attention has focused on messenger RNA (mRNA) as a potential therapeutic tool for enhancing protein synthesis [13]. Key modifications, such as *N*^6^-methyladenosine [14], 5-methylcytosine [15], and inosine [16, 17], have been well studied, particularly in the context of mRNA. However, uridine modifications have emerged as promising modifications in mRNA therapeutics due to their ability to modulate immune responses when incorporated into RNA [18–20]. Modifications like isomerization [21], thiolation [22], reduction [23], and methylation [24] of uridine lead to chemically distinct modifications that have potential medical applications, including reducing proinflammatory responses.

**Figure 1.**
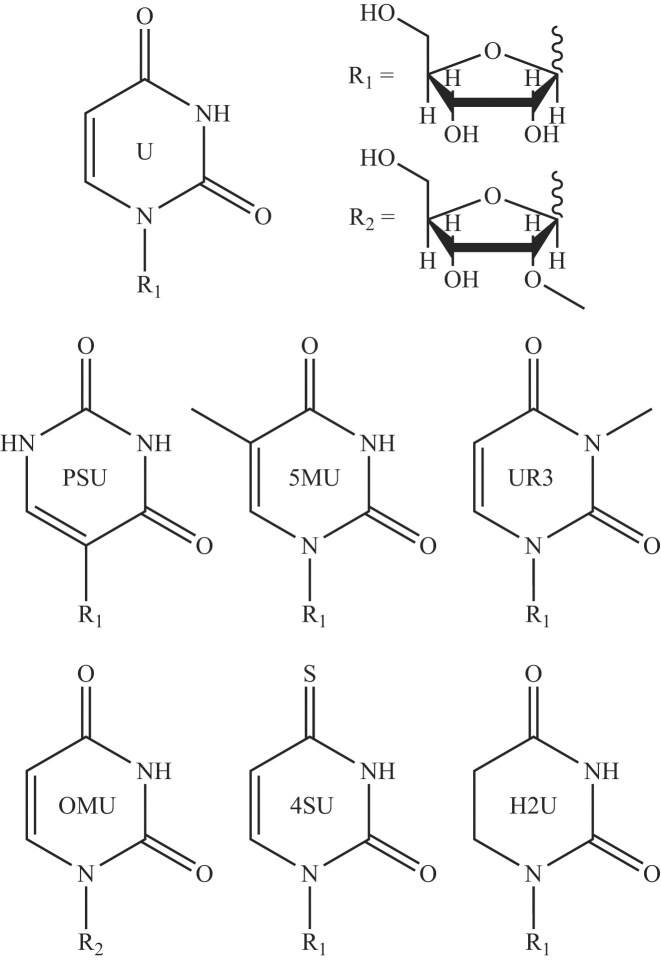
Structures of uridine and its nucleotide modifications. The canonical uridine base (U) is shown at the top left, which can be linked to either ribose (R1) or O2′-methylribose (R2), both shown at the top right. Below, the uridine modifications analyzed in this study are depicted: pseudouridine (PSU), 5-methyluridine (5MU), 3-methyluridine (UR3), O2′-methyluridine (OMU), 4-thiouridine (4SU), and 5,6-dihydrouridine (H2U). Each structure is labeled with its corresponding three-character RCSB PDB identifier, shown centrally on each base.

Among the uridine modifications, pseudouridine (PSU) is the most prevalent naturally occurring RNA modification, found in ~0.2%–6% of mammalian mRNA [21, 25]. PSU is formed through an enzymatically mediated process, creating a unique C1′–C5 glycosidic linkage after rotation along the N3-C6 axis (Fig. 1). This modification has been challenging to synthesize in high yields, but recent advances in semi-enzymatic synthesis pathways have made it more accessible [26]. Incorporation of PSU into RNA oligonucleotides has been shown to stabilize RNA duplexes significantly, increasing stability by up to 1.7 kcal/mol compared to U [27]. The effect of PSU incorporation is strongly dependent on the local structural context; in some noncanonical motifs, it can stabilize interactions, but in others, it may destabilize them [28]. When used as a therapeutic, RNA molecules containing PSU residues have been observed to evade immune response, enhance stability, and regulate protein expression [29, 30]. These findings support the incorporation of methylated PSU in mRNA vaccines as a strategy to prevent diseases such as severe acute respiratory syndrome coronavirus 2 (SARS-CoV-2) [29]. Although PSU modifications are known to occur at specific sites within RNA, quantitative techniques for mapping PSU in RNA have only recently been developed [31, 32]. To date, there has been no systematic or quantitative analysis of PSU, or any other U modifications, in previously solved RNA 3D structures.

In addition to PSU, several other modified uridine modifications (Fig. 1), including 5MU, have been shown to reduce innate immune recognition of RNA, thereby lowering cytokine expression when incorporated into transcripts [20]. 5MU is a naturally occurring byproduct of pyrimidine catabolism [33], and specific methyltransferases catalyze the conversion of U to 5MU at defined RNA sites [34]. Although 5MU does not alter the Watson–Crick–Franklin (WCF) base pairing face (Fig. 1), its presence at the wobble position of codon–anticodon interactions has been associated with reduced translation efficiency [35, 36]. This effect is thought to arise from electronic differences between the nucleobases of U and 5MU, potentially weakening hydrogen bonding along the WCF face [35]. The influence of 5MU on RNA stability is context-dependent, with evidence suggesting it can either stabilize or destabilize RNA depending on its position within the molecule [35, 37]. In comparison to PSU, 5MU is found at significantly lower levels in mammalian systems, typically between ~0.001% and 0.0059% in mRNA of various human cell types and mouse tissues [38], although its abundance can reach up to 1% in certain plant species [39]. Another relatively abundant uridine modification is 3-methyluridine (UR3), a methylated modification formed through the action of specific methyltransferases that convert U to UR3. This modification plays an important role in rRNA function [40, 41]. Notably, UR3 has been shown to disrupt triple helix formation [42], likely due to the disruption of hydrogen bonding on the WCF face.

Other uridine modifications, such as 4-thiouridine (4SU) and O2'-methyluridine (OMU), have also been shown to enhance RNA stability and protect against nuclease degradation [24, 43–46]. Notably, 4SU has been widely adopted in chemical biology as a biocompatible analog for RNA labeling and tracking [47–49]. However, while generally stabilizing, 4SU incorporation in tRNA can lead to UV sensitivity and growth arrest in some prokaryotes [47]. Methylated nucleotides, such as OMU, are essential for the biogenesis and function of human mitochondrial ribosomes [50], and their presence in mRNA has also been linked to increased transcript stability [51].

In contrast to the stabilizing effects of PSU, 5MU, UR3, OMU, and 4SU, the uridine modification 5,6-dihydrouridine (H2U) undergoes enzymatic reduction that disrupts aromaticity, leading to increased local flexibility within RNA structures [52, 53]. H2U is excreted in human urine and has been identified as a biomarker of RNA degradation [54]. Elevated levels of H2U have also been associated with certain cancers [55], and it is reported to be the second most abundant modification in tRNA after PSU [55, 56]. While these modifications are well characterized in terms of their chemical structures and biochemical pathways, their specific structural and functional consequences within native RNA 3D structures remain poorly understood. This represents a significant knowledge gap, particularly given the growing interest in RNA modifications as therapeutic targets and tools.

The Research Collaboratory for Structural Bioinformatics Protein Data Bank (RCSB PDB) provides a valuable resource for studying biological macromolecules in three-dimensional detail [57]. In recent years, advances in cryogenic electron microscopy (cryo-EM) have positioned the technique as one of the primary tools for elucidating the 3D structures of biological macromolecules [58]. Additionally, the third generation of AlphaFold [59–62] and new RoseTTA methods [63] have contributed to a rapid increase in the number of computed structure models (CSMs) of proteins available in the RCSB PDB. While CSMs provide insights comparable to low-resolution structures, they often fail to fully account for heteromolecular interactions, such as those between proteins and nucleic acids. High-confidence structures, however, are typically derived from nuclear magnetic resonance (NMR), cryo-EM, or X-ray diffraction techniques [64]. These high-resolution structures have been valuable in investigating RNA’s structural features and identifying recurring patterns, which can be used to make predictions [65–67].

Despite the wealth of structural data now available, no systematic study has yet explored the spatial distribution, conformational properties, and structural consequences of uridine modification in solved RNA structures. To address this gap, the current work investigates common uridine modifications, specifically PSU, 5MU, UR3, OMU, 4SU, and H2U, within the RCSB PDB. We aim to identify their locations, characterize their conformational preferences, and assess their local structural impact relative to unmodified uridine residues. These insights provide a foundation for understanding how RNA modifications influence structure and function and may guide future efforts in RNA 3D structure prediction and RNA-based therapeutic design.

## Materials and methods

### Database mapping and structure fetching

The RCSB PDB is a comprehensive repository of 3D biological macromolecular structures [68]. In RNA structures, modified residues (those deviating from the canonical A, C, G, or U nucleotides) are typically represented by a three-letter code that uniquely identifies the modification in the structure file. To examine common RNA modifications, we selected the six most frequently occurring uridine modifications in the RCSB PDB, each chosen for their distinct base and/or sugar modifications, as well as their demonstrated biological significance. These modifications include PSU, 5MU, UR3, OMU, 4SU, and H2U. These modifications represent various types, such as methylation of the base and/or sugar, oxidation of the base, and isomerization of the base. Structural representations of these modified nucleotides are depicted in Fig. 1.

**Figure 2.**
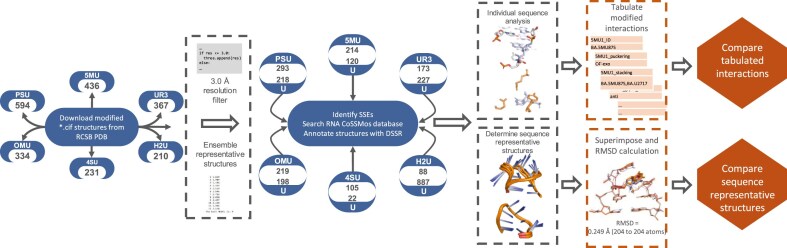
Workflow for structure identification, annotation, and comparison of uridine modifications. Each step in the workflow was conducted independently for structures containing uridine modifications and their corresponding unmodified (uridine-containing) counterparts. Secondary structure elements (SSEs) containing uridine modifications were identified and annotated using Dissecting the Spatial Structure of RNA (DSSR). Corresponding SSEs containing canonical uridine were identified via RNA Characterization of Secondary Structure Motifs (CoSSMos) and also annotated using DSSR. Sequence-representative structures (SRSs) were then superimposed, and all-atom RMSD values were calculated to assess structural differences. Base interactions within sequence-representative SSEs were analyzed and compared, followed by a comprehensive comparison of interactions across all identified SSEs.

Structures containing one of the six uridine modifications were retrieved from the RCSB PDB in the PDBX/mmCIF (*.cif) file format [69]. All structures containing the RNA-type uridine modification of interest were identified using the RCSB PDB Ligand Expo (ligand-expo.rcsb.org). This analysis relies on annotations present in the PDB structures at the time of extraction, specifically the modified nucleotide identifiers as submitted by the original depositors. An overview of the entire workflow is provided in Fig. 2.

### Sorting criteria and dataset refinement

Structures were initially sorted according to the identity of the modified nucleotide. To ensure data quality, structures solved by X-ray crystallography and cryo-EM with a resolution >3.0 Å were removed from the dataset. The relationship between the quality of structural data and the fidelity of derived molecular models remains poorly characterized [70]. While higher-resolution structures generally provide more reliable detail, we selected a 3.0 Å resolution cutoff to balance structural quality with dataset size. A more stringent threshold would have significantly reduced the number of structures available for analysis. Notably, a 3.0 Å resolution is widely accepted in the field as sufficient for assessing overall RNA conformation and base pairing geometry, especially in large RNA molecules, for which high-resolution structures remain relatively scarce [71–74]. Structures solved using NMR are typically submitted as an ensemble of simulated lowest-energy conformations, each containing a distinct set of atomic coordinates. To reduce redundancy in these cases, a method similar to that described previously was applied [75]. The average structure for each NMR ensemble was calculated, and the ensemble structure with the lowest all-atom RMSD to this average structure was selected as the representative structure for the ensemble. Only these representative structures were used for secondary structure element (SSE) identification, characterization, and subsequent analysis.

### Identification and characterization of SSEs

RNA SSEs were identified and characterized using the software Disecting the Spatial Structure of RNA (DSSR) [76]. DSSR, an RNA-specific successor to 3DNA [77], was employed to analyze RNA structures containing U modifications. The analysis was performed using default parameters, and all output data were saved in a *.json file format. For each *.json file, relevant information regarding the U modification residues was extracted. This information included the type of SSE, its associated nucleotide sequence, hydrogen bond acceptor/donor groups, base stacking (π stacking) interactions, sugar pucker, and glycosidic angle. The distance cutoff for identifying hydrogen bonding and base stacking interactions was set to 4.0 Å, as per DSSR’s default settings. Additionally, Python-based web-scraping was used to extract metadata for each RNA structure, including the molecule type (e.g. tRNA, mRNA, rRNA) and its organism of origin. The frequency of each interaction type was calculated and compared both within individual SSEs and between different SSEs.

### Uridine-containing structure retrieval

Following the identification of SSEs containing U modifications, sequences of these SSEs (including the first closing base pairs) were used to search the RNA Characterization of Secondary Structure Motifs (CoSSMos) database [75] for corresponding structures containing U in place of the U modifications. All modified nucleotides were reverted to their canonical counterparts (A, C, G, or U) prior to the search. A list of modified residues with their corresponding canonical counterparts is provided in Supplementary Table S1. A resolution cutoff of 3.0 Å was applied for structures solved using X-ray diffraction and cryo-EM. For NMR structures, only the ensemble representative structures were included in the analysis. The retrieved structure data were downloaded in a *.txt tab-delimited file format. SSEs were then extracted from their parent PDB structures using an established protocol [65]. Following extractions, the structural quality of each SSE was assessed according to standard protocols [65, 66]. In brief, structures were screened to ensure all atoms were present, each atom had a single coordinate, and all H atoms were removed.

### Identification of sequence representative structures

In order to address redundancy in the dataset, SRSs were determined for all structures sharing the same SSE and sequence, following a previously established protocol [65]. In brief, each structure containing the same SSE was superimposed and averaged. An all-atom RMSD calculation was then performed to compare each individual structure to the average structure. The structure with the lowest all-atom RMSD to the average structure was designated as the sequence-representative structures (SRS). Subsequently, all SRSs were annotated using DSSR.

### Comparison of uridine-containing and uridine modification-containing SRSs

Uridine-containing structures were analyzed as described above. Structures and features of the modified RNAs were compared to their unmodified counterparts.

## Results and discussion

### Occurrences of uridine modifications in solved RNA 3D structures

The frequency of occurrence for each uridine modification was initially determined without restrictions on the dataset. A query of the RCSB PDB database yielded 594, 436, 367, 334, 231, and 210 structure files containing at least one PSU, 5MU, UR3, OMU, 4SU, or H2U residue, respectively (Table 1). The majority of these structures were solved by X-ray crystallography for PSU and by cryo-EM for 5MU, UR3, OMU, 4SU, and H2U. To refine the dataset, we applied a resolution cutoff of ≤3.0 Å and selected ensemble representative structures for those solved via NMR. While structures with resolutions of ≤2.5 Å are generally considered more reliable for confidently assigning features such as sugar pucker and torsion angles [78, 79], using this threshold as a cutoff would have considerably limited the size of our dataset. An additional 21 structures were excluded from the analysis due to missing atoms in the RCSB PDB entries, DSSR processing issues, incomplete SSEs, or the inability to clip the SSEs (Supplementary Table S2). Following these criteria, the refined dataset included 293, 214, 173, 219, 105, and 88 structures containing at least one PSU, 5MU, UR3, OMU, 4SU, or H2U residue, respectively (Table 1). In total, 2982 PSU residues were identified, making it the most frequently occurring modification in this dataset. 5MU was the second most frequent with 736 occurrences, followed by OMU (429), 4SU (314), UR3 (232), and H2U (171), ranking third through sixth in abundance, respectively (Table 1). It is likely that the true extent of modifications is underreported, owing to historical constraints in model-building and annotation practices, particularly in early ribosome structures.

**Table 1.** Frequency of structures with each modification and the total number of residues in the final dataset

Modification ^b^	Entire dataset				Dataset for analysis ^a^				
	X-ray diffraction	Cryo-electron microscopy	Nuclear magnetic resonance ^c^	Total structures	X-ray diffraction	Cryo-electron microscopy	Nuclear magnetic resonance ^c^	Total structures	Total residues ^d^
pseudouridine (PSU)	301	275	18	594	158	117	18	293	2982
5-methyluridine (5MU)	184	251	1	436	108	105	1	214	736
3-methyluridine (UR3)	183	184	0	367	91	82	0	173	232
O2'-methyluridine (OMU)	115	214	5	334	95	119	5	219	429
4-thiouridine (4SU)	110	121	0	231	64	41	0	105	314
5,6-dihydrouridine (H2U)	78	131	1	210	42	45	1	88	171

a Refinement includes restricting X-ray diffraction and electron microscopy structures to those with a resolution ≤3.0 Å.

b The RCSB PDB three-letter codes for each modification are in parentheses.

c Frequencies count only the ensemble representative structures.

d Total number of modified residues in the refined dataset. Structures can have more than one modified residue.

### Distribution of uridine modifications across RNA types and SSEs

**Figure 3.**
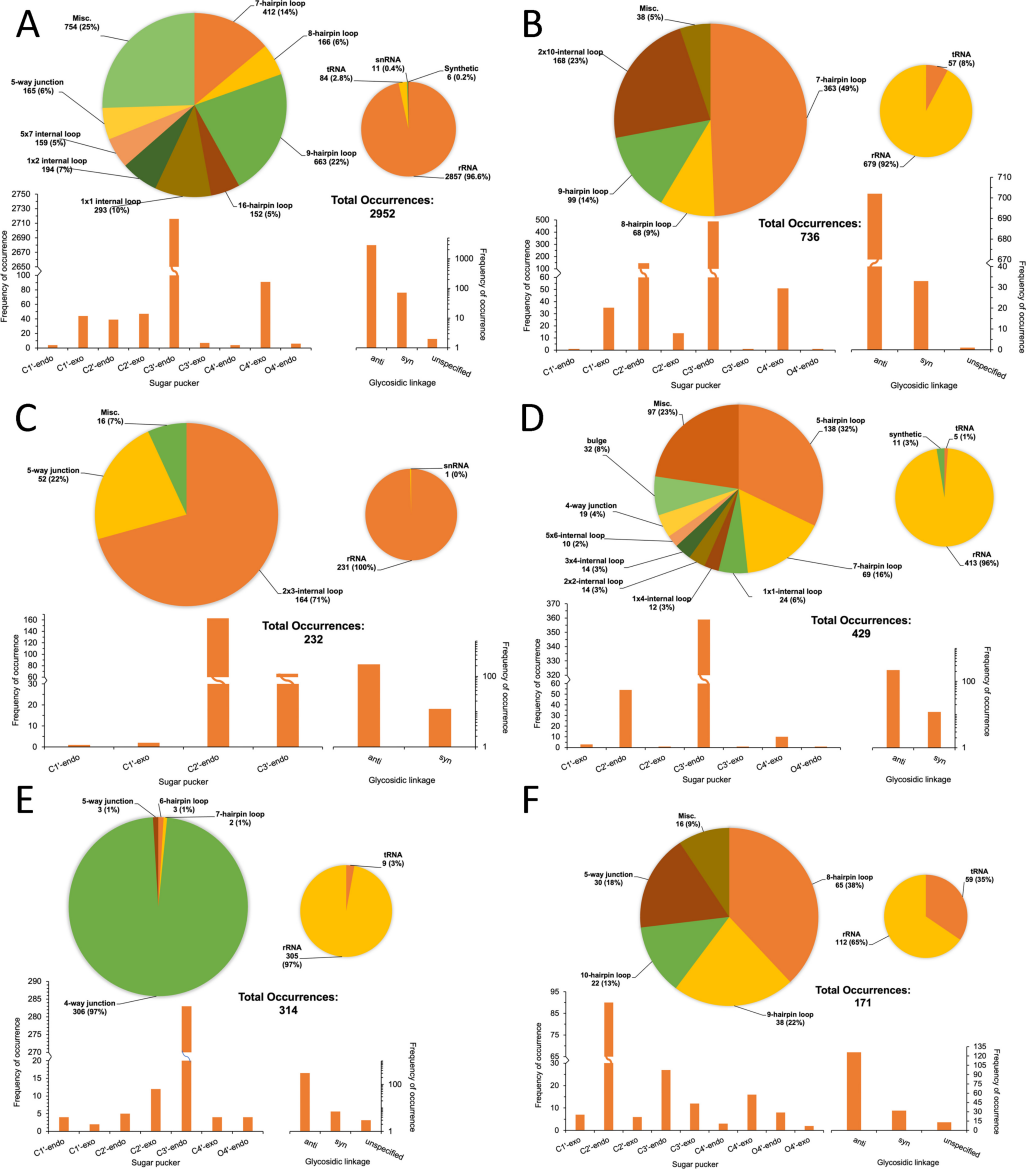
Distribution of uridine modifications in RNA structures from the RCSB PDB. The occurrence and structural context of (**A**) PSU, (**B**) 5MU, (**C**) OMU, (**D**) 4SU, (**E**) UR3, and (**F**) H2U are shown. Total occurrences represent the number of times each modification appears in *.cif structure files with resolution ≤3.0 Å. Multiple modifications may be present within a single file. Large pie charts indicate the distribution of modifications across SSEs, while small pie charts show their distribution among annotated RNA types. The bottom left panel presents the sugar pucker conformations for each modification, and the bottom right panel shows the corresponding glycosidic linkage distributions. The “miscellaneous” category includes SSE types that individually account for <1% of the dataset; collectively, this category may exceed 1%.

The distribution of uridine modifications in solved RNA structures was analyzed by RNA type and SSE. Across all modifications, the highest frequency of modifications was observed in rRNA (Fig. 3). PSU was found broadly in rRNA, tRNA, snRNA, and synthetic constructs (Fig. 3A), consistent with its known presence across multiple RNA types at conserved pseudouridylation sites [21]. 5MU was also predominantly observed in rRNA (92% of occurrences), with the remaining ~8% detected in tRNA (Fig. 3B). In tRNA, both PSU and 5MU commonly localize to the conserved TΨC loop [80], a hairpin motif. Like PSU, 5MU is associated with specific methylation sites in diverse RNA classes [37, 81]. UR3 was unique in that it was not detected in tRNA. This was unexpected given previous reports of UR3 modifications in certain tRNAs [82]. Instead, UR3 was primarily found in rRNA (Fig. 3C). OMU and 4SU were also strongly enriched in rRNA, accounting for 96% and 97% of total occurrences, respectively (Fig. 3D and E). OMU was also found in synthetic constructs (~3% of cases) (Fig. 3D), while 4SU was occasionally observed in other RNA types. The location of methylated modifications (5MU, OMU, and UR3) is consistent with the known roles in rRNA function and translational regulation [83]. Finally, H2U showed a distinctive distribution, with the highest proportion (35%) occurring in tRNA (Fig. 3F), aligning with recent findings of H2U across various RNA species, including mRNA [84].

Analysis of SSRs revealed that uridine modifications were most commonly located in hairpin loops, with variation by modification type. About 52% of PSU residues were found in hairpins, predominantly of seven, eight, and nine unpaired nucleotides (Fig. 3A). Similarly, 5MU was enriched in hairpins of these sizes, accounting for 72% of its total occurrences (Fig. 3B). While the TΨC loop in tRNA typically forms a pentaloop [85], variants such as heptaloops were also observed in the dataset (Supplementary Table S3). In contrast, UR3 showed the lowest frequency in hairpins (<5%) and is clustered in the less-common SSE category “Misc” (Fig. 3C). OMU was frequently observed in five- or seven-nucleotide hairpins (48% of occurrences; Fig. 3D). 4SU was more commonly associated with junctions, though six- and seven-nucleotide hairpins were also observed (Fig. 3E). Notably, 76% of H2U residues occurred within hairpin structures (Fig. 3F), including sizes of 8, 9, and 10 nucleotides, as well as rare loop sizes grouped into the Misc. category.

Due to the predominance of uridine modifications within hairpin SSEs and the sequence redundancy observed in these regions, subsequent structural analyses by SRSs and comparisons to unmodified Us were restricted to hairpin motifs. Other SSEs, such as junctions and internal loops, had limited representation and lacked sufficient unmodified equivalents in the RNA CoSSMos database [75].

### Glycosidic bond angles and sugar puckers of uridine modifications

The glycosidic bond in nucleotides typically forms between the N9 atom of purine or the N1 atom of pyrimidine nucleobases and the C1′ atom of the ribose sugar. In PSU residues, however, the linkage occurs between the C5 atom of the nucleobase and the C1′ of the ribose. The glycosidic torsion angle (χ) defines the orientation of the base relative to the sugar: *syn* conformations are defined by χ angles between +90° and −90°, while *anti*-conformations fall outside this range [86]. In pyrimidine nucleotides, the O2 atom (or O4 in PSU) plays a key role in sterically favoring the *anti*-conformation, with the base oriented away from the sugar to minimize clashes. Consistent with this, >80% of the uridine modifications analyzed adopted an *anti*-glycosidic conformation (Fig. 3). The remaining conformations were predominantly *syn*, with <1% categorized as “unspecified” (Fig. 3). These unspecified confirmations reflect cases where DSSR could not assign a definitive glycosidic angle during structural annotation.

The ribose sugar pucker significantly influences RNA structure, particularly through its effect on base pairing and hydrogen-bonding interactions [87]. The five torsional angles of the ribose ring can be simplified into discrete puckering conformations, such as C2′-*endo* (S-type) and C3′-*endo* (N-type), which provide insight into RNA secondary structure [88, 89]. Among PSU residues, the C3′-*endo* pucker, characteristic of A-form RNA helices, was most prevalent, followed by C4′-*exo* (Fig. 3A). This was a surprising result, as puckering involving C1′, C4′, and O4′ atoms is generally considered unfavorable [88]. Additionally, C-glycoside nucleosides, such as PSU, often favor a higher population of C2′-*endo* puckers due to a diminished anomeric effect [90]. The majority of 5MU, OMU, and 4SU residues also exhibited a predominant C3′-*endo* sugar pucker (Fig. 3B–E). These results align with prior observations that incorporation of 4SU into RNA favors the A-form-like C3′-*endo* conformation [91]. In 5MU and OMU, the second most common pucker was C2′-*endo* (Fig. 3B and D).

In contrast, C2′-*endo* sugar puckers, more typical of B-form structures [92], were the dominant conformation in UR3 and H2U residues (Fig. 3C and F). This observation is consistent with previous studies [55, 93]. For H2U, the saturation of the C5 = C6 double bond (relative to unmodified U) stabilizes the C2′-*endo* conformation by up to 1.5 kcal/mol [53]. Likewise, UR3 has been shown to favor the C2′-*endo* conformation, with prior work reporting up to 45% prevalence [93]. In our analysis, ~70% of UR3 residues adopted this conformation (Fig. 3C). In summary, PSU, 5MU, OMU, and 4SU modifications predominantly favor sugar puckers characteristic of A-form RNA, while UR3 and H2U modifications preferentially adopt B-form-like C2′-*endo* conformations. These trends reflect structural adaptations driven by both steric and electronic effects specific to each modification.

### Comparison of modified and unmodified hairpin SRSs

As previously noted, uridine modifications were predominantly found within hairpin structures. Therefore, only hairpins containing these modified residues were selected for analysis using SRSs. To assess structural impacts of the modifications, we also generated SRSs for the corresponding unmodified sequences, where each modified residue was substituted with its canonical uridine equivalent (Supplementary Table S3). Definitions and abbreviations for modifications and their canonical counterparts are provided in Supplementary Table S1. SRSs capture consensus conformations for recurrent sequence-structure motifs across multiple RNA structures, effectively reducing redundancy while preserving biologically relevant features [65, 66]. For comparative analysis, only sequences with both modified and unmodified counterparts available in the RNA CoSSMos database [75] were included.

The number of unique hairpin sequences varied by modification type: 32 PSU-containing sequences (with 19 unmodified counterparts), 14 for 5MU (8 unmodified), 1 for UR3 (1 unmodified), 14 for OMU (12 unmodified), and 2 each for 4SU and H2U (2 unmodified counterparts each) (Supplementary Table S3). In several instances, multiple modified variants mapped to a single unmodified sequence due to positional heterogeneity or multiple modifications at a single site. Unmodified structures were notably more prevalent than modified ones across all categories.

Structural similarity between modified and unmodified SRSs was assessed via all-atom RMSD. An RMSD threshold of $ \le $1.0 Å was used to define similarity, consistent with prior studies of RNA structural families [65, 66, 94]. SRS pairs exceeding this threshold were considered structurally distinct. The majority of SRSs for each uridine modification occupied similar Cartesian space when compared to the uridine equivalents, having RMSD values $ \le $1.0 Å.

*PSU*. Approximately 53% of PSU-modified SRSs were structurally similar to their unmodified counterparts (Supplementary Fig. S1A, C, D, G–I, K, L, N, O, Q, R, and X–AB), while the rest deviated significantly (RMSD > 1.0 Å; Supplementary Fig. S1B, E, F, J, M, P, S–W, and AC–AF). Although PSU retains a canonical WCF face, it introduces unique interaction potentials, via its N1 imino proton. This proton can participate in hydrogen bonding networks with the phosphate backbone [27, 28, 95, 96]. While direct interactions involving bridging water molecules were not consistently resolved, <10% of PSU residues formed N1 hydrogen bonds, suggesting such interactions may be transient or occluded.

**Figure 4.**
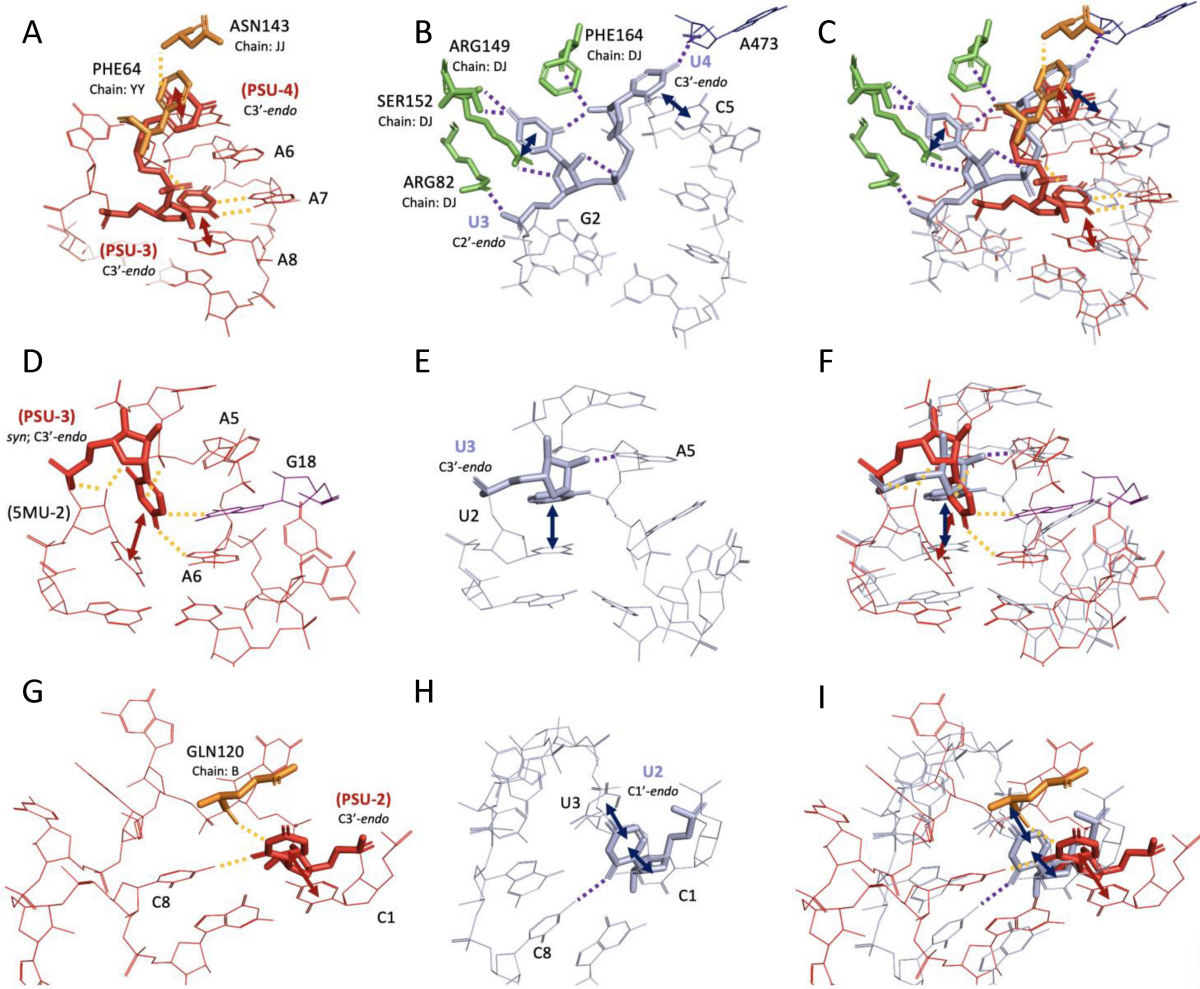
Structural comparisons of SRS heptaloops containing PSU versus unmodified uridine. Each row presents a pairwise comparison between modified and unmodified structures, followed by their structure superposition. First row: Heptaloops from (**A**) PSU-containing structure 7UCK_UGPPCAAAG_9_820 and (**B**) unmodified structure 6ZQC_UGUUCAAAG_D3_765, with (**C**) their superimposition. Second row: Heptaloops from (**D**) PSU-containing structure 6V3A_GtPCAAGUC_v_54 and (**E**) unmodified structure 3OV7_GUUCAAGUC_D_13, with (**F**) their superimposition. Third row: Heptaloops from (**G**) PSU-containing structure 1ASZ_CPUGUCgCG_S_631 and (**H**) unmodified structure 3J16_CUUGUCGCG_L_32, with (**I**) their superimposition. Stacking interactions are indicated by double-headed arrows, and hydrogen bonds are shown as dashed lines. The sugar pucker conformation for residues of interest is noted for each residue. Unless specified otherwise, glycosidic linkages are assumed to be in the *anti-*conformation. Amino acid residues are labeled using the three-letter code followed by the original residue index and chain ID. Tertiary nucleic acid residues are labeled by their original index and are part of the same chain as the SSE.

Examples (Fig. 4) illustrate how PSU affects base pairing, stacking, and tertiary contacts, sometimes inducing conformational flips or enabling noncanonical hydrogen bonds, such as *syn* glycosidic angles facilitating tertiary interactions (e.g. kissing loop contacts). Comparison of the heptaloop SRS interactions between structures 7UCK_UGPPCAAAG_9_820 (Fig. 4A) and 6ZQC_UGUUCAAAG_D3_765 (Fig. 4B) reveals key differences driven by the presence of PSU. In 7UCK, the N1 imino proton of PSU-3 forms a hydrogen bond with the phosphate backbone of residue A6 within the same SSE, an interaction not possible in the unmodified structure. In contrast, the corresponding unmodified residue in 6ZQC is flipped away from the hairpin SSE and interacts with two arginine residues, a serine, and adjacent uridine bases (Fig. 4B). Additionally, π-stacking occurs between one of the arginine side chains and the nucleobase. PSU-3 also engages in Hoogsteen base pairing with A7 and stacks with A8, further stabilizing the heptaloop (Fig. 4A). PSU-4 exhibits favorable stacking with a nearby phenylalanine and forms hydrogen bonds with the backbone of an asparagine residue. The corresponding unmodified residue in 6ZQC, while still forming a hydrogen bond with the phenylalanine, stacks with the C5 residue of the SSE and hydrogen bonds with residue A473 from the same chain (Fig. 4B). These conformational and interaction differences are clearly illustrated in the superimposed structures (Fig. 4C and Supplementary Fig. S1J).

A similar trend is observed when comparing 6V3A_GtPCAAGUC_v_54 (Fig. 4D) and 3OV7_GUUCAAGUC_D_13 (Fig. 4E). In 6V3A, PSU-3 adopts a *syn* conformation, enabling its N1 imino proton to form a tertiary hydrogen bond with G18 from the same chain, an example of a kissing loop interaction commonly seen in tRNAs [97]. The unmodified residue in 3OV7 remains in the *anti-*conformation and lacks such tertiary interactions (Fig. 4E). These distinctions become more apparent in the overlaid structures (Fig. 4F and Supplementary Fig. S1P).

The comparison between 1ASZ_CPUGUCgCG_S_631 (Fig. 4G) and 3J16_CUUGUCGCG_L_32 SRSs (Fig. 4H) reveals the highest RMSD among the SRS pairs analyzed (Supplementary Fig. S1AF), yet the local interactions remain largely conserved. Both the modified and unmodified residues stack with C1 in the closing base pair of the hairpin loop and form hydrogen bonds with C8 in the same SSE. However, PSU has an additional tertiary hydrogen bond with a nearby glutamine residue that is absent in the unmodified structure. Conversely, the unmodified residue adopts a C1′-*endo* sugar pucker, allowing enhanced stacking with neighboring U3 (Fig. 4H). These structural and conformational differences are further emphasized in the superimposed models (Fig. 4I and Supplementary Fig. S1AF).

**Figure 5.**
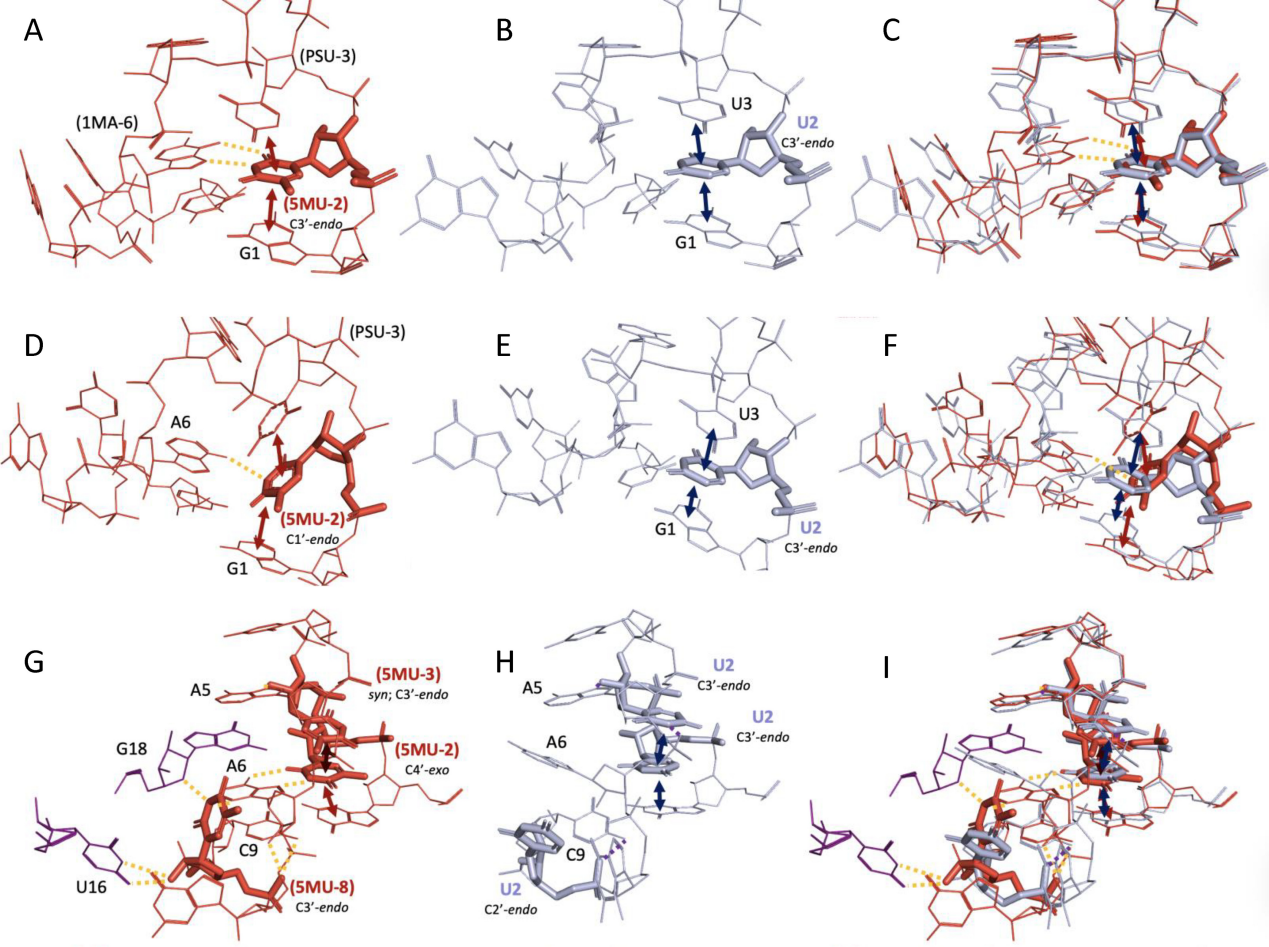
Structural comparisons of SRS heptaloops containing 5MU versus unmodified uridine. Each row presents a pairwise comparison between modified and unmodified structures, followed by their structure superposition. First row: Heptaloops from (**A**) 5MU-containing structure 1F7V_GtPCAaGUC_B_953 and (**B**) unmodified structure 3OV7_GUUCAAGUC_D_13, with (**C**) their superimposition. Second row: Heptaloops from (**D**) 5MU-containing structure 6V3A_GtPCAAGUC_v_54 and (**E**) unmodified structure 3OV7_GUUCAAGUC_D_13, with (**F**) their superimposition. Third row: Heptaloops from (**G**) 5MU-containing structure 7OT5_GttCAAGtC_z_62 and (**H**) unmodified structure 3OV7_GUUCAAGUC_D_13, with (**I**) their superimposition. Stacking interactions are indicated by double-headed arrows, and hydrogen bonds are shown as dashed lines. The sugar pucker conformation for residues of interest is noted for each residue. Unless specified otherwise, glycosidic linkages are assumed to be in the anti conformation. Amino acid residues are labeled using the three-letter code followed by the original residue index and chain ID. Tertiary nucleic acid residues are labeled by their original index and are part of the same chain as the SSE.

*5MU*. Approximately 64% of 5MU-containing SRSs were structurally similar to their unmodified counterparts (Supplementary Fig. S2A–E, G, and L–N), while the remainder showed large deviations (RMSD > 1.7 Å; Supplementary Fig. S2F and H–K). The 5-methyl group, though preserving the WCF face, may restrict base rotation and influence sugar pucker. Comparative examples (Fig. 5) show how 5MU affects stacking, affects Hoogsteen pairing, and introduces steric constraints, particularly when adopting C1′-*endo* or *syn* conformations. The structures 1F7V_GtPCAaGUC_B_953, 6V3A_GtPCAAGUC_v_54, and 7OT5_GttCAAGtC_z_62 SRSs were compared to the same unmodified SRS, 3OV7_GUUCAAGUC_D_13 (Fig. 5), which also appears in Fig. 4E and F for consistency. In the 1F7V_GtPCAaGUC_B_953 structure, the 5MU modification stacks with adjacent G1 and PSU-3 residues and forms a Hoogsteen base pair with residue 1MA-6 (Fig. 5A). In contrast, the corresponding unmodified SRS only exhibits base stacking interactions (Fig. 5B). An overlay of the two structures highlights these differences (Fig. 5C). The 6V3A_GtPCAAGUC_v_54 structure, also shown in Fig. 4D and F, contains a 5MU residue adopting an unfavorable C1′-*endo* sugar pucker. Despite this, it maintains stacking interactions with the *syn-*oriented PSU-3 and G1 residues (Fig. 5D). A single hydrogen bond is formed with A6 within the same SSE. The unmodified counterpart shows similar stacking interactions (Fig. 5E), and the structural overlays reveal the shifted position of the 5MU base (Fig. 5F). The 7OT5_GttCAAGtC_z_62 SRS contains three 5MU modifications (Fig. 5G). One of these adopts a C4′-*exo* sugar pucker and forms Hoogsteen base pairing with A6, along with stacking interactions with flanking residues. Like the PSU in 6V3A, this 5MU is also in the *syn* conformation. The third 5MU, located in the hairpin region, engages in hydrogen bonding with residues U16 and G18 outside of the SSE, as well as with C9 from the closing base pair (Fig. 5G). In the unmodified structure, the first three residues in the hairpin engage in similar stacking interactions (Fig. 5H). The final residue analyzed within the SSE showed the greatest conformational variability, as illustrated in the overlay (Fig. 5I).

**Figure 6.**
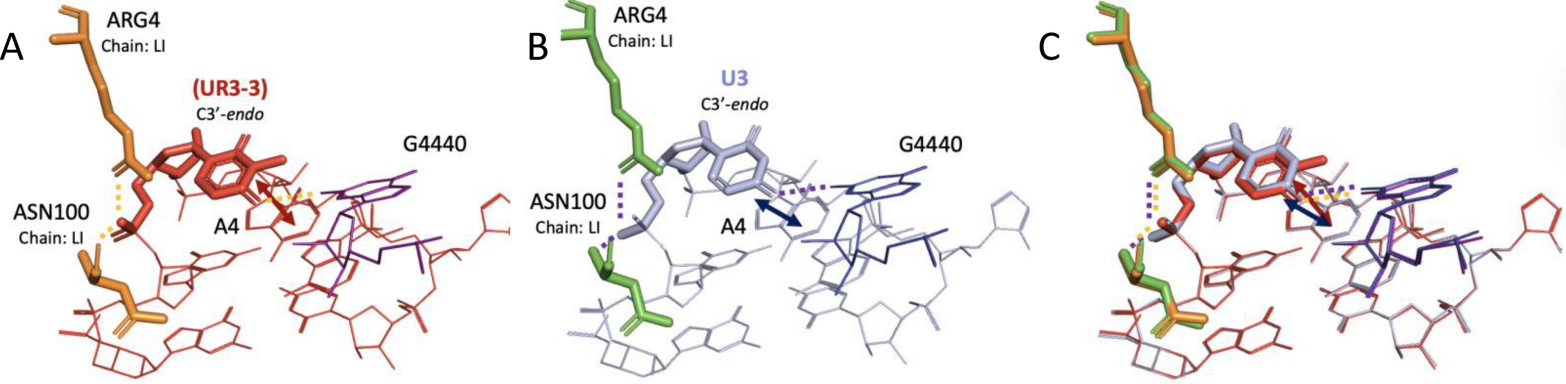
Structural comparisons of SRS pentaloops containing UR3 versus unmodified uridine. The row presents a pairwise comparison between modified and unmodified structures, followed by their structure superposition. Pentaloops from (**A**) UR3-containing structure 7F5S_GGuAAGC_L5_1864 and (**B**) unmodified structure 6Y6X_GGUAAGC_L5_1865, with (**C**) their superimposition. Stacking interactions are indicated by double-headed arrows, and hydrogen bonds are shown as dashed lines. The sugar pucker conformation for residues of interest is noted for each residue. Unless specified otherwise, glycosidic linkages are assumed to be in the anti conformation. Amino acid residues are labeled using the three-letter code followed by the original residue index and chain ID. Tertiary nucleic acid residues are labeled by their original index and are part of the same chain as the SSE.

*UR3*. Structural comparison between the single SRSs revealed high similarity, with an RMSD $ \le $0.2 Å (Supplementary Fig. S3). Analysis of the pentaloop region in the modified 7F5S_GGuAAGC_L5_1864 (Fig. 6A) and unmodified 6Y6X_GGUAAGC_L5_1865 (Fig. 6B) structures showed that both maintain similar backbone hydrogen bonding interactions with nearby arginine and asparagine residues. Additionally, both structures exhibit base stacking with the adjacent A4 residue within the same SSE, as well as tertiary hydrogen bonding interactions with the G4440 residue on the same chain (Fig. 6A and B). Importantly, the spatial arrangement of the nucleobases in both the modified and unmodified SRSs does not permit hydrogen bonding involving the N3 atom. As a result, the methyl group at position 3 in the modified structure does not disrupt any potential interactions. The structural similarities are further confirmed by the overlay shown in Fig. 6C and Supplementary Fig. S3.

**Figure 7.**
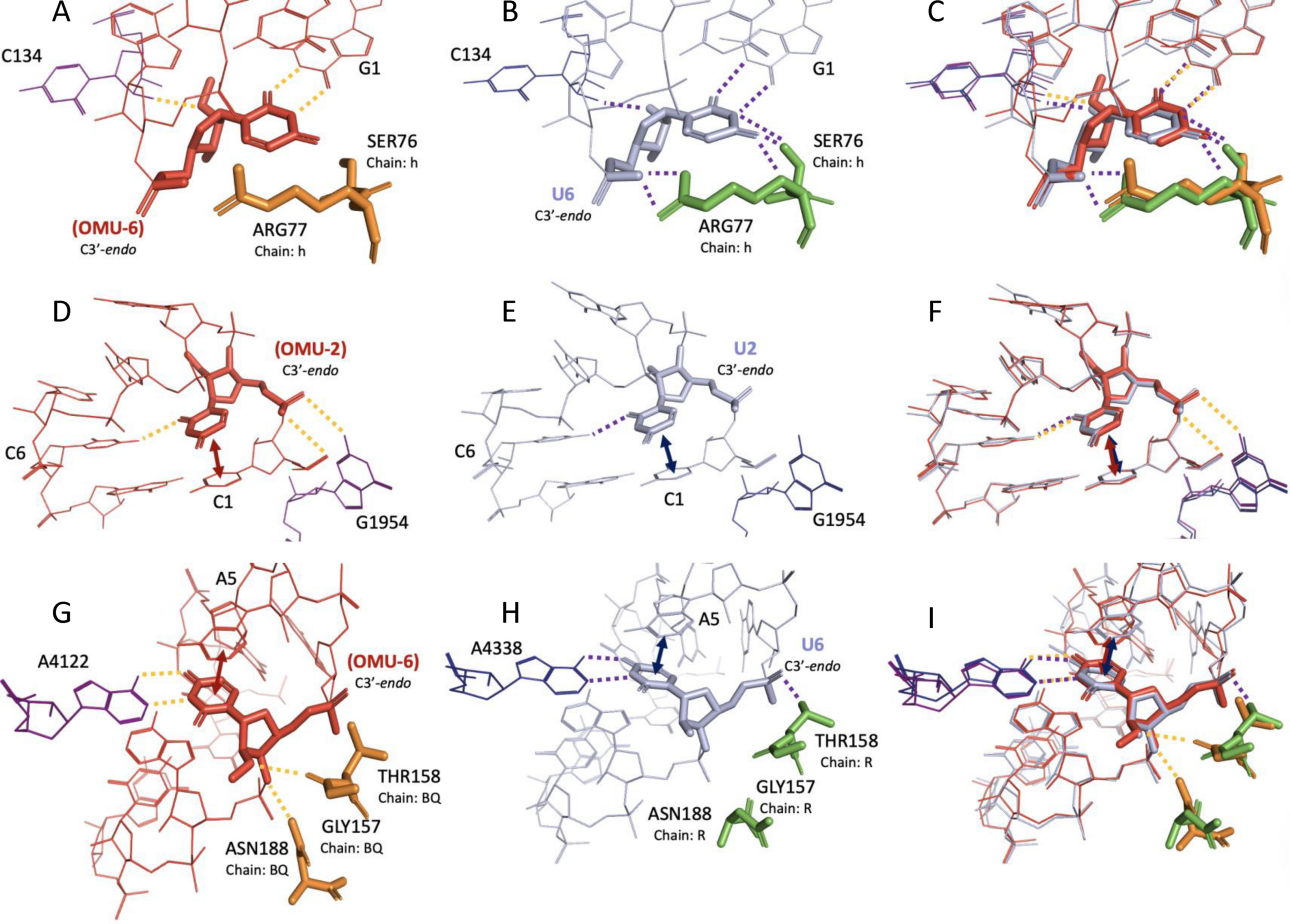
Structural comparisons of SRS hairpin loops containing OMU versus unmodified uridine. Each row presents a pairwise comparison between modified and unmodified structures, followed by their structure superposition. First row: Tetraloop from (**A**) OMU-containing structure 6AZ3_GGUAAu_3_5 and (**B**) unmodified structure 5T2A_GGUAAU_E_6, with (**C**) their superimposition. Second row: Pentaloops from (**D**) OMU-containing structure 7RQ8_CuGUUCG_1A_2551 and (**E**) unmodified structure 7ZOD_CUGUUCG_b_2552, with (**F**) their superimposition. Third row: Heptaloops from (**G**) OMU-containing structure 7O7Z_UUCAGuACG_B5_4047 and (**H**) unmodified structure 6OM7_UUCAGUACG_t_4264, with (**I**) their superimposition. Stacking interactions are indicated by double-headed arrows, and hydrogen bonds are shown as dashed lines. The sugar pucker conformation for residues of interest is noted for each residue. Unless specified otherwise, glycosidic linkages are assumed to be in the anti conformation. Amino acid residues are labeled using the three-letter code followed by the original residue index and chain ID. Tertiary nucleic acid residues are labeled by their original index and are part of the same chain as the SSE.

*OMU*. All OMU-modified SRSs were structurally similar to their unmodified equivalents (maximum RMSD was 0.835 Å; Supplementary Fig. S4). OMU uniquely modifies the ribose (at the O2′ position), rather than the nucleobase (Fig. 1). As anticipated, this modification had minimal impact on base pairing or stacking, although subtle differences in tertiary hydrogen bonding patterns were observed (Fig. 7). Notably, OMU sometimes enabled or disrupted interactions with nearby protein residues, depending on local geometry. To compare OMU and U as closing base pairs in tetraloops, we analyzed the structural features of several SRS. In 6AZ3_GGUAAu_3_5 (Fig. 7A) and 5T2A_GGUAAU_E_6 (Fig. 7B), both structures exhibit canonical G-U hydrogen bonding interactions along the WCF face. Additionally, both the modified OMU and unmodified U residues form a tertiary hydrogen bond with C134 in the same chain via the O2′ atom. This interaction is maintained even in the presence of the 2′-O-methyl group in OMU-containing structure. However, the unmodified U residue engages in additional hydrogen bonds with nearby arginine and serine residues (Fig. 7B), which are absent in the OMU structure (Fig. 7A). Subtle conformational differences that may explain the loss of these interactions in the OMU variant are apparent in the overlaid structures (Fig. 7C and Supplementary Fig. S4B).

A similar comparison between 7RQ8_CuGUUCG_1A_2551 (Fig. 7D) and 7ZOD_CUGUUCG_b_2552 (Fig. 7E) highlights further distinctions. In 7RQ8, the OMU-2 residue participates in tertiary hydrogen bonding with G1954, stacks with and forms hydrogen bonds with C1 in the same SSE, and also forms a single hydrogen bond with C6 (Fig. 7D). In contrast, the unmodified U2 residue in 7ZOD lacks the tertiary and C1 hydrogen bonding interactions, despite the proximity of G1954 (Fig. 7E). These conformational differences are also illustrated in the structural overlay (Fig. 7F and Supplementary Fig. S4D).

In the case of 7O7Z_UUCAGuACG_B5_4047 (Fig. 7G) and 6OM7_UUCAGUACG_t_4264 (Fig. 7H), OMU-6 engages in a tertiary hydrogen bond with an adenine residue that mimics a standard WCF interaction, along with stacking interactions with another adenine within the same SSE. Remarkably, despite the presence of the O2′-methyl group, OMU-6 also forms hydrogen bonds with nearby glycine and asparagine residues (Fig. 7G). The unmodified U6 residue maintains the same WCF base pairing and stacking interactions but lacks hydrogen bonds with the glycine and asparagine. Instead, it forms a backbone-mediated hydrogen bond with a threonine residue, a contact not observed in the OMU structure (Fig. 7H). The distinct spatial arrangement of glycine and asparagine favors hydrogen bonding with OMU-6 but not with U6, as illustrated in the overlaid structures (Fig. 7I and Supplementary Fig. S4M).

**Figure 8.**
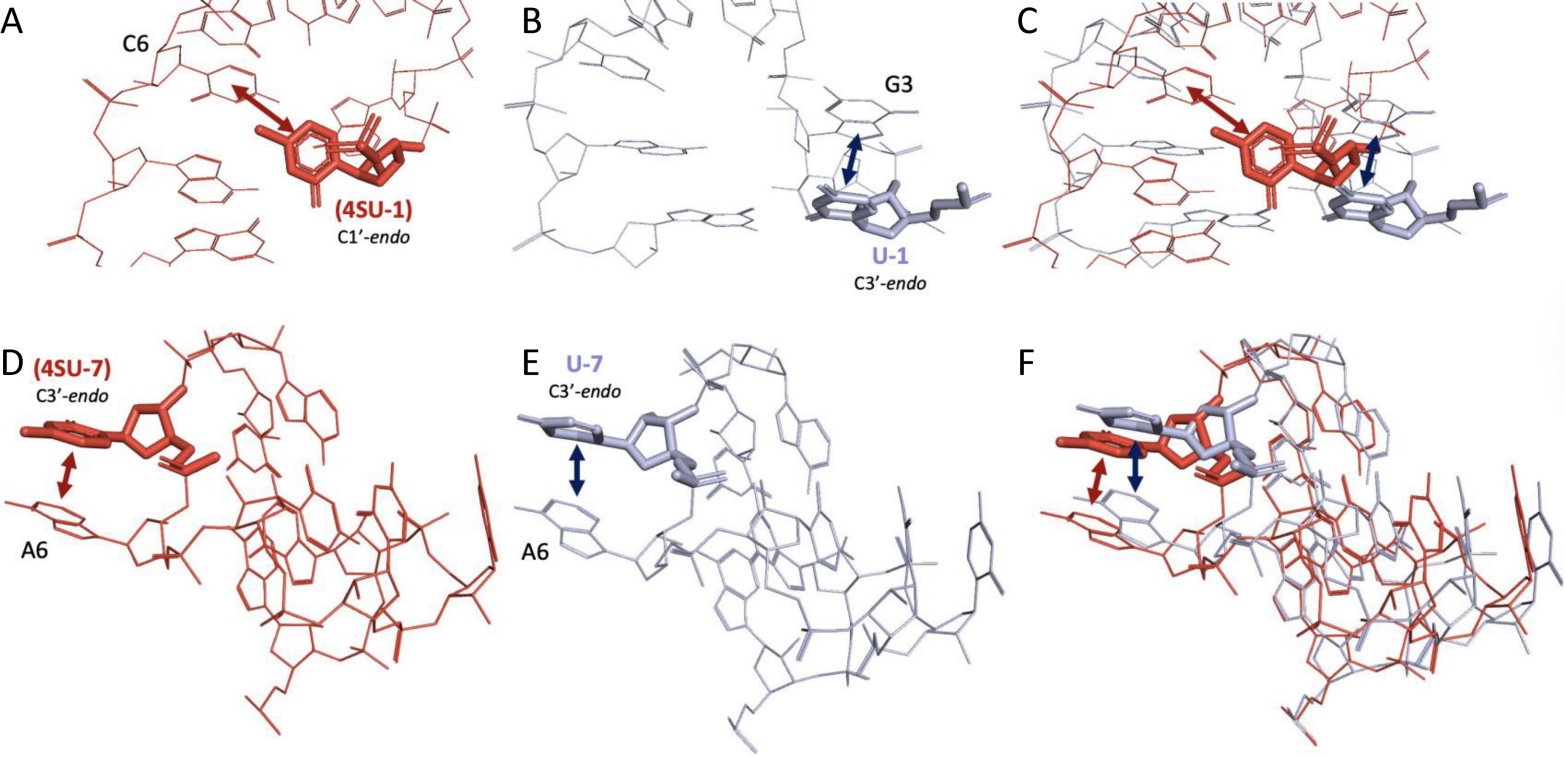
Structural comparisons of SRS hairpin loops containing 4SU versus unmodified uridine. Each row presents a pairwise comparison between modified and unmodified structures, followed by their structure superposition. First row: Hexaloops from (**A**) 4SU-containing structure 5WDT_uGGAGCAG_w_8 and (**B**) unmodified structure 7MT2_UGGAGCAG_A_2767, with (**C**) their superimposition. Second row: Heptaloops from (**D**) 4SU-containing structure 6SKF_AGCCCAuAU_BA_2575 and (**E**) unmodified structure 4V4N_AGCCCAUAU_A1_2587, with (**F**) their superimposition. Stacking interactions are indicated by double-headed arrows, and hydrogen bonds are shown as dashed lines. The sugar pucker conformation for residues of interest is noted for each residue. Unless specified otherwise, glycosidic linkages are assumed to be in the anti conformation. Amino acid residues are labeled using the three-letter code followed by the original residue index and chain ID. Tertiary nucleic acid residues are labeled by their original index and are part of the same chain as the SSE.

*4SU*. Among two 4SU-containing SRSs, one was structurally similar (RMSD of ≈ 0.8 Å), while the other showed a substantial shift (RMSD ≈ 2.8 Å; Supplementary Fig. S5). In the 5WDT_uGGAGCAG_w_8 (Fig. 8A) and 7MT2_UGGAGCAG_A_2767 (Fig. 8B) SRSs, the 4SU adopts a C1′-*endo* sugar pucker, a rare and typically unfavorable conformation. This structural deviation repositions the modified nucleotide to promote stacking interactions with the C6 residue in the loop. In contrast, the unmodified uridine in 7MT2 adopts the canonical C3′-*endo* conformation and does not participate in similar stacking interactions (Fig. 8B). The extent of this conformational shift is clearly illustrated in the overlayed structures (Fig. 8C).

A similar pattern is observed in the 6SKF_AGCCCAuAU_BA_2575 (Fig. 8D) and 4V4N_AGCCCAUAU_A1_2587 (Fig. 8E) SRSs, where the 4SU-7 and U7 residues show comparable local interactions and conformational features. These structural similarities are highlighted in the overlay (Fig. 8F). Despite the presence of the 4-thio substitution, no hydrogen bonding interactions were detected involving the O4 atom of uridine or the S4 atom of 4SU in any of the SRSs analyzed.

**Figure 9.**
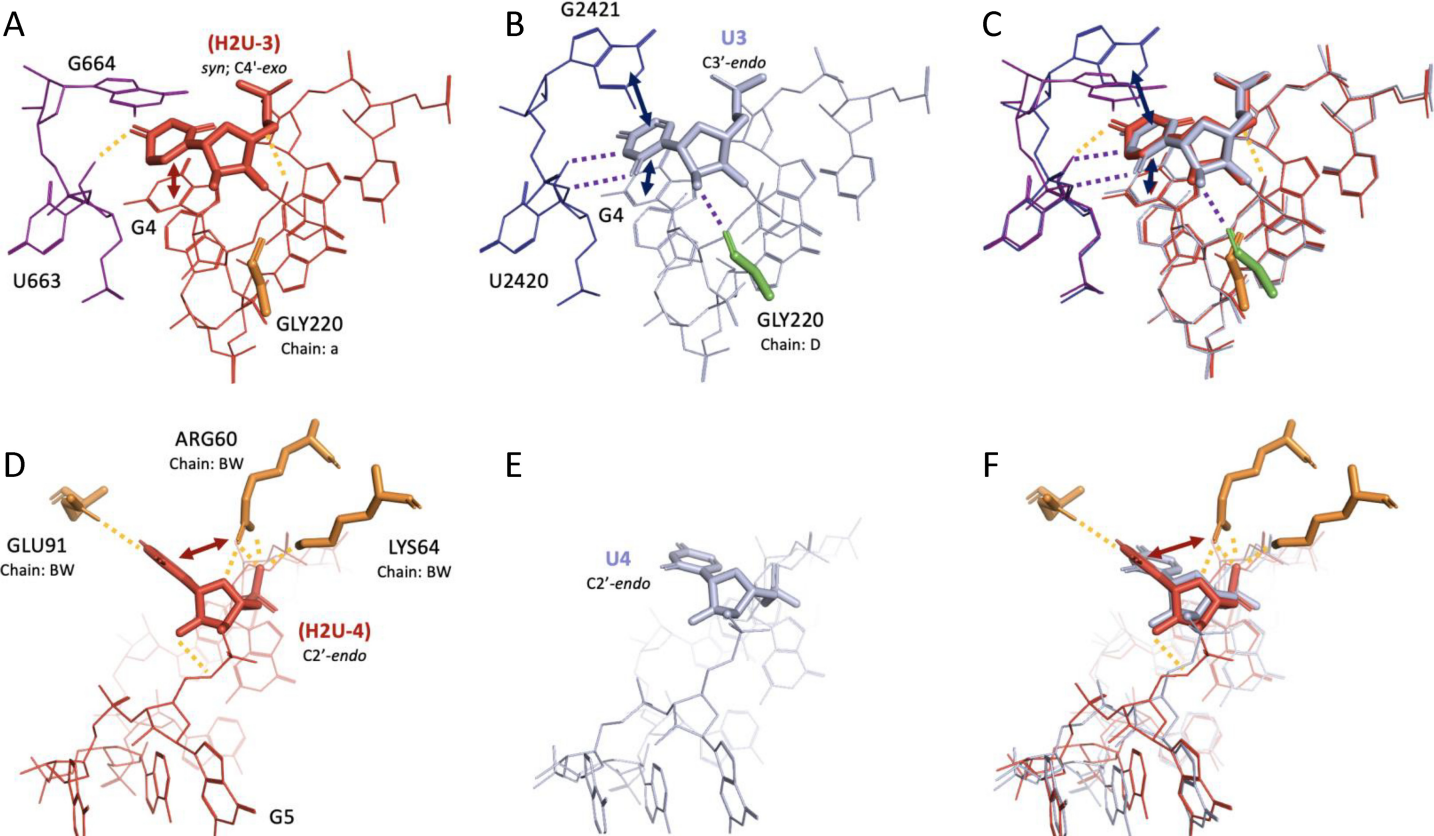
Structural comparisons of SRS hairpin loops containing 5,6-dihydrouridine versus unmodified uridine. Each row presents a pairwise comparison between modified and unmodified structures, followed by their structure superposition. First row: Heptaloops from (**A**) H2U-containing structure 3JCS_CGuGAG_2_1402 and (**B**) unmodified structure 7QIW_CGUGAG_2_2968, with (**C**) their superimposition. Second row: Heptaloops from (**D**) H2U-containing structure 6TB3_CAGuGGAAG_n_13 and (**E**) unmodified structure 7NRD_CAGUGGAAG_Sn_14, with (**F**) their superimposition. Stacking interactions are indicated by double-headed arrows, and hydrogen bonds are shown as dashed lines. The sugar pucker conformation for residues of interest is noted for each residue. Unless specified otherwise, glycosidic linkages are assumed to be in the anti conformation. Amino acid residues are labeled using the three-letter code followed by the original residue index and chain ID. Tertiary nucleic acid residues are labeled by their original index and are part of the same chain as the SSE.

*H2U*. Both H2U-modified SRSs showed high similarity (RMSD ≈ 0.3 and 0.8 Å; Supplementary Fig. S6), despite the saturation of the C5 = C6 bond. In some cases, H2U adopted *syn* glycosidic angles and engaged in distinct hydrogen bonding or stacking interactions not observed in unmodified structures (Fig. 9). Comparison of the 3JCS_CGuGAG_2_1402 (Fig. 9A) and 7QIW_CGUGAG_2_2968 (Fig. 9B) SRSs reveals that the H2U-3 residue adopts a *syn* glycosidic conformation and a C4′-*exo* sugar pucker. Despite these deviations from the canonical geometry, H2U-3 is still able to form a single hydrogen bond with U663 and maintains stacking interactions with the adjacent G4 residue within the same SSE (Fig. 9A). Notably, this occurs even with the C5 = C6 bond in the uracil ring being saturated due to the H2U modification. In contrast, the unmodified U3 residue in 7QIW adopts a standard anti glycosidic conformation and forms multiple hydrogen bonds, specifically with U2420 in the same chain and the backbone carbonyl of a nearby glycine residue (Fig. 9B). These differences in hydrogen bonding and base positioning are evident in the overlaid structures (Fig. 9C and Supplementary Fig. S6A), where the shift in the position of the H2U-3 O2′ atom and the glycine carbonyl likely prevent the formation of these interactions. Interestingly, while H2U exhibits some unique interactions, it does not clearly surpass U in hydrogen bonding capacity in this context.

Further structural comparison of 6TB3_CAGuGGAAG_n_13 (Fig. 9D) and 7NRD_CAGUGGAAG_Sn_14 (Fig. 9E) shows a more pronounced impact of the H2U modification. Here, H2U-4 forms several hydrogen bonds with surrounding glutamic acid, arginine, and lysine residues and engages in stacking interactions with the arginine side chain (Fig. 9D). In contrast, the corresponding U4 residue in 7NRD exhibits no detectable interactions (Fig. 9E). These conformational distinctions are highlighted in the structural overlay (Fig. 9F and Supplementary Fig. S6B).

In summary, most uridine modifications preserved local hairpin structure, with SRSs of modified residues frequently matching those of their unmodified counterparts. However, notable deviations were observed for PSU, 5MU, and 4SU in certain sequence contexts. These differences were often attributable to steric or electronic effects introduced by the modifications, which could impact sugar pucker, base stacking, or hydrogen bonding patterns. Despite these changes, many modified sequences retained similar interaction profiles, emphasizing the nuanced yet context-dependent structural consequences of uridine modifications in RNA.

### Applications to RNA structure prediction and development

In this study, six of the most frequently observed uridine modifications in the RCSB PDB were systematically analyzed. These modifications encompass a diverse range of chemical alterations, including changes to the WCF face, isomerization, thiolation, and ribose modifications (Fig. 1). Known modification sites were accurately identified across the dataset, underscoring the reliability and robustness of the proposed workflow (Fig. 2). While most SRSs containing U-modifications adopted conformations closely resembling those of their unmodified counterparts, several modified uridines engaged in distinct, non-canonical interactions not typically observed in unmodified RNA. This work offers a detailed structural characterization of uridine modifications, highlighting their distribution, conformational behavior, and potential structural impact. These findings provide a foundational framework for advancing RNA structure prediction models and inform the rational design of RNA-based therapeutics and function studies involving post-transcriptional modifications.

## Supplementary Material

lqaf197_Supplemental_File

## Data Availability

All structure files utilized in this analysis are available in the RCSB PDB. All data associated with the analysis of these structures are available in the article and in its online Supplementary data.

## References

[1] Moore LD, Le T, Fan G. DNA methylation and its basic function. Neuropsychopharmacol. 2013;38:23–38. 10.1038/npp.2012.112. PMC3521964 22781841

[2] Nabel CS, Lee JW, Wang LC et al. Nucleic acid determinants for selective deamination of DNA over RNA by activation-induced deaminase. Proc Natl Acad Sci USA. 2013;110:14225–30. 10.1073/pnas.1306345110. 23942124 PMC3761612

[3] Tanaka M, Chock PB. Oxidative modifications of RNA and its potential roles in biosystem. Front Mol Biosci. 2021;8:685331. 10.3389/fmolb.2021.685331. 34055897 PMC8149912

[4] Esteller M . Cancer epigenomics: DNA methylomes and histone-modification maps. Nat Rev Genet. 2007;8:286–98. 10.1038/nrg2005. 17339880

[5] Barbieri I, Kouzarides T. Role of RNA modifications in cancer. Nat Rev Cancer. 2020;20:303–22. 10.1038/s41568-020-0253-2. 32300195

[6] Shi H, Chai P, Jia R et al. Novel insight into the regulatory roles of diverse RNA modifications: re-defining the bridge between transcription and translation. Mol Cancer. 2020;19:78. 10.1186/s12943-020-01194-6. 32303268 PMC7164178

[7] Pan T . Modifications and functional genomics of human transfer RNA. Cell Res. 2018;28:395–404. 10.1038/s41422-018-0013-y. 29463900 PMC5939049

[8] Martinez NM, Su A, Burns MC et al. Pseudouridine synthases modify human pre-mRNA co-transcriptionally and affect pre-mRNA processing. Mol Cell. 2022;82:645–59. 10.1016/j.molcel.2021.12.023. 35051350 PMC8859966

[9] Boccaletto P, Stefaniak F, Ray A et al. MODOMICS: a database of RNA modification pathways. 2021 update. Nucleic Acids Res. 2022;50:D231–5. 10.1093/nar/gkab1083. 34893873 PMC8728126

[10] Boccaletto P, Machnicka MA, Purta E et al. MODOMICS: a database of RNA modification pathways. 2017 update. Nucleic Acids Res. 2018;46:D303–7. 10.1093/nar/gkx1030. 29106616 PMC5753262

[11] Dunin-Horkawicz S, Czerwoniec A, Gajda MJ et al. MODOMICS: a database of RNA modification pathways. Nucleic Acids Res. 2006;34:D145–9. 10.1093/nar/gkj084. 16381833 PMC1347447

[12] Holley RW, Everett GA, Madison JT et al. Nucleotide sequences in the yeast alanine transfer ribonucleic acid. J Biol Chem. 1965;240:2122–8. 10.1016/S0021-9258(18)97435-1. 14299636

[13] Kariko K . Modified uridines are the key to a successful message. Nat Rev Immunol. 2021;21:619. 10.1038/s41577-021-00608-w. 34580453

[14] Perry RP, Kelley DE. Existence of methylated messenger RNA in mouse L cells. Cell. 1974;1:37–42. 10.1016/0092-8674(74)90153-6.

[15] Squires JE, Patel HR, Nousch M et al. Widespread occurrence of 5-methylcytosine in human coding and non-coding RNA. Nucleic Acids Res. 2012;40:5023–33. 10.1093/nar/gks144. 22344696 PMC3367185

[16] Jaffrey SR . An expanding universe of mRNA modifications. Nat Struct Mol Biol. 2014;21:945–6. 10.1038/nsmb.2911. 25372308 PMC4384663

[17] Paul MS, Bass BL. Inosine exists in mRNA at tissue-specific levels and is most abundant in brain mRNA. EMBO J. 1998;17:1120–7. 10.1093/emboj/17.4.1120. 9463389 PMC1170460

[18] Moradian H, Roch T, Anthofer L et al. Chemical modification of uridine modulates mRNA-mediated proinflammatory and antiviral response in primary human macrophages. Mol Ther Nucleic Acids. 2022;27:854–69. 10.1016/j.omtn.2022.01.004. 35141046 PMC8807976

[19] Wilkinson E, Cui YH, He YY. Roles of RNA modifications in diverse cellular functions. Front Cell Dev Biol. 2022;10:828683. 10.3389/fcell.2022.828683. 35350378 PMC8957929

[20] Kariko K, Buckstein M, Ni H et al. Suppression of RNA recognition by Toll-like receptors: the impact of nucleoside modification and the evolutionary origin of RNA. Immunity. 2005;23:165–75. 10.1016/j.immuni.2005.06.008. 16111635

[21] Charette M, Gray MW. Pseudouridine in RNA: what, where, how, and why. IUBMB Life. 2000;49:341–51. 10.1080/152165400410182. 10902565

[22] Zheng YY, Wu Y, Begley TJ et al. Sulfur modification in natural RNA and therapeutic oligonucleotides. RSC Chem Biol. 2021;2:990–1003. 10.1039/D1CB00038A. 34458821 PMC8341892

[23] Yu F, Tanaka Y, Yamashita K et al. Molecular basis of dihydrouridine formation on tRNA. Proc Natl Acad Sci USA. 2011;108:19593–8. 10.1073/pnas.1112352108. 22123979 PMC3241823

[24] Kotelawala L, Grayhack EJ, Phizicky EM. Identification of yeast tRNA Um_44_ 2′-O-methyltransferase (Trm44) and demonstration of a Trm44 role in sustaining levels of specific tRNA^Ser^ species. RNA. 2008;14:158–69. 10.1261/rna.811008. 18025252 PMC2151035

[25] Li X, Zhu P, Ma S et al. Chemical pulldown reveals dynamic pseudouridylation of the mammalian transcriptome. Nat Chem Biol. 2015;11:592–7. 10.1038/nchembio.1836. 26075521

[26] Riley AT, Sanford TC, Woodard AM et al. Semi-enzymatic synthesis of pseudouridine. Bioorg Med Chem Lett. 2021;44:128105. 10.1016/j.bmcl.2021.128105. 33991631

[27] Hudson GA, Bloomingdale RJ, Znosko BM. Thermodynamic contribution and nearest-neighbor parameters of pseudouridine-adenosine base pairs in oligoribonucleotides. RNA. 2013;19:1474–82. 10.1261/rna.039610.113. 24062573 PMC3851715

[28] Vogele J, Duchardt-Ferner E, Kruse H et al. Structural and dynamic effects of pseudouridine modifications on noncanonical interactions in RNA. RNA. 2023;29:790–807. 10.1261/rna.079506.122. 36868785 PMC10187676

[29] Nelson J, Sorensen EW, Mintri S et al. Impact of mRNA chemistry and manufacturing process on innate immune activation. Sci Adv. 2020;6:eaaz6893. 10.1126/sciadv.aaz6893. 32637598 PMC7314518

[30] Mauger DM, Cabral BJ, Presnyak V et al. mRNA structure regulates protein expression through changes in functional half-life. Proc Natl Acad Sci USA. 2019;116:24075–83. 10.1073/pnas.1908052116. 31712433 PMC6883848

[31] Yarian CS, Basti MM, Cain RJ et al. Structural and functional roles of the N1- and N3-protons of Ψ at tRNA’s position 39. Nucleic Acids Res. 1999;27:3543–9. 10.1093/nar/27.17.3543. 10446245 PMC148599

[32] Marchand V, Pichot F, Neybecker P et al. HydraPsiSeq: a method for systematic and quantitative mapping of pseudouridines in RNA. Nucleic Acids Res. 2020;48:e110. 10.1093/nar/gkaa769. 32976574 PMC7641733

[33] Gao S, Sun Y, Chen X et al. Pyrimidine catabolism is required to prevent the accumulation of 5-methyluridine in RNA. Nucleic Acids Res. 2023;51:7451–64. 10.1093/nar/gkad529. 37334828 PMC10415118

[34] Madsen CT, Mengel-Jorgensen J, Kirpekar F et al. Identifying the methyltransferases for m^5^U747 and m^5^U1939 in 23S rRNA using MALDI mass spectrometry. Nucleic Acids Res. 2003;31:4738–46. 10.1093/nar/gkg657. 12907714 PMC169892

[35] Jones JD, Franco MK, Smith TJ et al. Methylated guanosine and uridine modifications in *S. cerevisiae* mRNAs modulate translation elongation. RSC Chem Biol. 2023;4:363–78. 10.1039/D2CB00229A. 37181630 PMC10170649

[36] Jones JD, Franco MK, Giles RN et al. Conserved 5-methyluridine tRNA modification modulates ribosome translocation. Proc Natl Acad Sci USA. 2024;121:e2401743121. 10.1073/pnas.2401743121. 39159370 PMC11363252

[37] Auxilien S, Rasmussen A, Rose S et al. Specificity shifts in the rRNA and tRNA nucleotide targets of archaeal and bacterial m5U methyltransferases. RNA. 2011;17:45–53. 10.1261/rna.2323411. 21051506 PMC3004065

[38] Cheng QY, Xiong J, Ma CJ et al. Chemical tagging for sensitive determination of uridine modifications in RNA. Chem Sci. 2020;11:1878–91. 10.1039/C9SC05094A. 34123281 PMC8148390

[39] Gao S, Sun Y, Chen X et al. Pyrimidine catabolism is required to prevent the accumulation of 5-methyluridine in RNA. Nucleic Acids Res. 2023;51:7451–64. 10.1093/nar/gkad529. 37334828 PMC10415118

[40] Basturea GN, Rudd KE, Deutscher MP. Identification and characterization of RsmE, the founding member of a new RNA base methyltransferase family. RNA. 2006;12:426–34. 10.1261/rna.2283106. 16431987 PMC1383581

[41] Sharma S, Yang J, Duttmann S et al. Identification of novel methyltransferases, Bmt5 and Bmt6, responsible for the m3U methylations of 25S rRNA in *Saccharomyces cerevisiae*. Nucleic Acids Res. 2014;42:3246–60. 10.1093/nar/gkt1281. 24335083 PMC3950682

[42] Kunkler CN, Schiefelbein GE, O’Leary NJ et al. A single natural RNA modification can destabilize a U•A-T-rich RNA•DNA–DNA triple helix. RNA. 2022;28:1172–84. 10.1261/rna.079244.122. 35820700 PMC9380742

[43] Belanger F, Stepinski J, Darzynkiewicz E et al. Characterization of hMTr1, a human Cap1 2′-O-ribose methyltransferase. J Biol Chem. 2010;285:33037–44. 10.1074/jbc.M110.155283. 20713356 PMC2963352

[44] Werner M, Purta E, Kaminska KH et al. 2′-O-ribose methylation of cap2 in human: function and evolution in a horizontally mobile family. Nucleic Acids Res. 2011; 39:4756–68. 10.1093/nar/gkr038. 21310715 PMC3113572

[45] Baserga SJ, Yang XD, Steitz JA. An intact Box C sequence in the U3 snRNA is required for binding of fibrillarin, the protein common to the major family of nucleolar snRNPs. EMBO J. 1991;10:2645–51. 10.1002/j.1460-2075.1991.tb07807.x. 1714385 PMC452965

[46] Hori H . Methylated nucleosides in tRNA and tRNA methyltransferases. Front Genet. 2014;5:144. 10.3389/fgene.2014.00144. 24904644 PMC4033218

[47] Mueller EG, Buck CJ, Palenchar PM et al. Identification of a gene involved in the generation of 4-thiouridine in tRNA. Nucleic Acids Res. 1998;26:2606–10. 10.1093/nar/26.11.2606. 9592144 PMC147624

[48] Kambampati R, Lauhon CT. Evidence for the transfer of sulfane sulfur from IscS to ThiI during the *in vitro* biosynthesis of 4-thiouridine in *Escherichia coli* tRNA. J Biol Chem. 2000;275:10727–30. 10.1074/jbc.275.15.10727. 10753862

[49] Ohashi S, Nakamura M, Acharyya S et al. Development and comparison of 4-thiouridine to cytidine base conversion reaction. ACS Omega. 2024;9:9300–8. 10.1021/acsomega.3c08516. 38434802 PMC10905967

[50] Rorbach J, Boesch P, Gammage PA et al. MRM2 and MRM3 are involved in biogenesis of the large subunit of the mitochondrial ribosome. MBoC. 2014;25:2542–55. 10.1091/mbc.e14-01-0014. 25009282 PMC4148245

[51] Li Y, Yi Y, Gao X et al. 2′-O-methylation at internal sites on mRNA promotes mRNA stability. Mol Cell. 2024;84:2320–2336.e6. 10.1016/j.molcel.2024.04.011. 38906115 PMC11196006

[52] Kasprzak JM, Czerwoniec A, Bujnicki JM. Molecular evolution of dihydrouridine synthases. BMC Bioinf. 2012;13:153. 10.1186/1471-2105-13-153. PMC3674756 22741570

[53] Dalluge JJ, Hashizume T, Sopchik AE et al. Conformational flexibility in RNA: the role of dihydrouridine. Nucleic Acids Res. 1996;24:1073–9. 10.1093/nar/24.6.1073. 8604341 PMC145759

[54] Topp H, Duden R, Schoch G. 5,6-Dihydrouridine: a marker ribonucleoside for determining whole body degradation rates of transfer RNA in man and rats. Clin Chim Acta. 1993;218:73–82. 10.1016/0009-8981(93)90223-Q. 8299222

[55] Finet O, Yague-Sanz C, Marchand F et al. The Dihydrouridine landscape from tRNA to mRNA: a perspective on synthesis, structural impact and function. RNA Biology. 2022;19:735–50. 10.1080/15476286.2022.2078094. 35638108 PMC9176250

[56] Machnicka MA, Olchowik A, Grosjean H et al. Distribution and frequencies of post-transcriptional modifications in tRNAs. RNA Biology. 2014;11:1619–29. 10.4161/15476286.2014.992273. 25611331 PMC4615829

[57] Zardecki C, Dutta S, Goodsell DS et al. RCSB Protein Data Bank: a resource for chemical, biochemical, and structural explorations of large and small biomolecules. J Chem Educ. 2016;93:569–75. 10.1021/acs.jchemed.5b00404.

[58] Burley SK, Berman HM, Bhikadiya C et al. Protein Data Bank: the single global archive for 3D macromolecular structure data. Nucleic Acids Res. 2019;47:D520–8. 10.1093/nar/gky949. 30357364 PMC6324056

[59] Jumper J, Evans R, Pritzel A et al. Highly accurate protein structure prediction with AlphaFold. Nature. 2021;596:583–9. 10.1038/s41586-021-03819-2. 34265844 PMC8371605

[60] Akdel M, Pires DEV, Pardo EP et al. A structural biology community assessment of AlphaFold2 applications. Nat Struct Mol Biol. 2022;29:1056–67. 10.1038/s41594-022-00849-w. 36344848 PMC9663297

[61] Varadi M, Anyango S, Deshpande M et al. AlphaFold Protein Structure Database: massively expanding the structural coverage of protein-sequence space with high-accuracy models. Nucleic Acids Res. 2022;50:D439–44. 10.1093/nar/gkab1061. 34791371 PMC8728224

[62] Abramson J, Adler J, Dunger J et al. Accurate structure prediction of biomolecular interactions with AlphaFold 3. Nature. 2024;630:493–500. 10.1038/s41586-024-07487-w. 38718835 PMC11168924

[63] Baek M, DiMaio F, Anishchenko I et al. Accurate prediction of protein structures and interactions using a three-track neural network. Science. 2021;373:871–6. 10.1126/science.abj8754. 34282049 PMC7612213

[64] Shao C, Bittrich S, Wang S et al. Assessing PDB macromolecular crystal structure confidence at the individual amino acid residue level. Structure. 2022;30:1385–94. 10.1016/j.str.2022.08.004. 36049478 PMC9547844

[65] Richardson KE, Adams MS, Kirkpatrick CC et al. Identification and characterization of new RNA tetraloop sequence families. Biochemistry. 2019;58:4809–20. 10.1021/acs.biochem.9b00535. 31714066 PMC7745247

[66] Saon MS, Kirkpatrick CC, Znosko BM. Identification and characterization of RNA pentaloop sequence families. NAR Genom Bioinform. 2023;5:lqac102. 10.1093/nargab/lqac102. 36632613 PMC9830547

[67] Chavali SS, Cavender CE, Mathews DH et al. Arginine forks are a widespread motif to recognize phosphate backbones and guanine nucleobases in the RNA major groove. J Am Chem Soc. 2020;142:19835–9. 10.1021/jacs.0c09689. 33170672 PMC7937406

[68] Burley SK, Bhikadiya C, Bi C et al. RCSB Protein Data Bank: powerful new tools for exploring 3D structures of biological macromolecules for basic and applied research and education in fundamental biology, biomedicine, biotechnology, bioengineering and energy sciences. Nucleic Acids Res. 2021;49:D437–51. 10.1093/nar/gkaa1038. 33211854 PMC7779003

[69] Westbrook JD, Young JY, Shao C et al. PDBx/mmCIF ecosystem: foundational semantic tools for structural biology. J Mol Biol. 2022;434:167599. 10.1016/j.jmb.2022.167599. 35460671 PMC10292674

[70] Karplus PA, Diederichs K. Linking crystallographic model and data quality. Science. 2012;336:1030–3. 10.1126/science.1218231. 22628654 PMC3457925

[71] Casanal A, Shakeel S, Passmore LA. Interpretation of medium resolution cryoEM maps of multi-protein complexes. Curr Opin Struct Biol. 2019;58:166–74. 10.1016/j.sbi.2019.06.009. 31362190 PMC6863432

[72] Ramirez-Aportela E, Maluenda D, Fonseca YC et al. FSC-Q: a CryoEM map-to-atomic model quality validation based on the local Fourier shell correlation. Nat Commun. 2021;12:42. 10.1038/s41467-020-20295-w. 33397925 PMC7782520

[73] Vonck J, Mills DJ. Advances in high-resolution cryo-EM of oligomeric enzymes. Curr Opin Struct Biol. 2017;46:48–54. 10.1016/j.sbi.2017.05.016. 28624735

[74] Terwilliger TC, Adams PD, Afonine PV et al. Cryo-EM map interpretation and protein model-building using iterative map segmentation. Protein Sci. 2020;29:87–99. 10.1002/pro.3740. 31599033 PMC6933853

[75] Richardson KE, Kirkpatrick CC, Znosko BM. RNA CoSSMos 2.0: an improved searchable database of secondary structure motifs in RNA three-dimensional structures. Database. 2020;2020:baz153. 10.1093/database/baz153. 31950189 PMC6966092

[76] Hanson RM, Lu XJ. DSSR-enhanced visualization of nucleic acid structures in Jmol. Nucleic Acids Res. 2017;45:W528–33. 10.1093/nar/gkx365. 28472503 PMC5570162

[77] Lu XJ, Olson WK. 3DNA: a software package for the analysis, rebuilding and visualization of three-dimensional nucleic acid structures. Nucleic Acids Res. 2003;31:5108–21. 10.1093/nar/gkg680. 12930962 PMC212791

[78] Gray JG, Case DA. Refinement of RNA structures using amber force fields. Cryst. 2021;11:771. 10.3390/cryst11070771. PMC8570558 34745655

[79] Murray LJ, Arendall WB, Richardson DC et al. RNA backbone is rotameric. Proc Natl Acad Sci USA. 2003;100:13904–9. 10.1073/pnas.1835769100. 14612579 PMC283519

[80] Shigi N, Suzuki T, Tamakoshi M et al. Conserved bases in the TΨC loop of tRNA are determinants for thermophile-specific 2-thiouridylation at position 54*. J Biol Chem. 2002;277:39128–35. 10.1074/jbc.M207323200. 12177072

[81] Kong Y, Mead EA, Fang G. Navigating the pitfalls of mapping DNA and RNA modifications. Nat Rev Genet. 2023;24:363–81. 10.1038/s41576-022-00559-5. 36653550 PMC10722219

[82] Kelley M, Uhran M, Herbert C et al. Abundances of transfer RNA modifications and transcriptional levels of tRNA-modifying enzymes are sex-associated in mosquitoes. Insect Biochem Mol Biol. 2022;143:103741. 10.1016/j.ibmb.2022.103741. 35181477 PMC9034435

[83] Monaco PL, Marcel V, Diaz JJ et al. 2′-O-methylation of ribosomal RNA: towards an epitranscriptomic control of translation?. Biomolecules. 2018;8:106. 10.3390/biom8040106. 30282949 PMC6316387

[84] Draycott AS, Schaening-Burgos C, Rojas-Duran MF et al. Transcriptome-wide mapping reveals a diverse dihydrouridine landscape including mRNA. PLoS Biol. 2022;20:e3001622. 10.1371/journal.pbio.3001622. 35609439 PMC9129914

[85] Chan CW, Chetnani B, Mondragon A. Structure and function of the T-loop structural motif in noncoding RNAs. WIREs RNA. 2013;4:507–22. 10.1002/wrna.1175. 23754657 PMC3748142

[86] Lu XJ, Bussemaker HJ, Olson WK. DSSR: an integrated software tool for dissecting the spatial structure of RNA. Nucleic Acids Res. 2015;43:e142. 10.1093/nar/gkv716. 26184874 PMC4666379

[87] Williams AA, Darwanto A, Theruvathu JA et al. Impact of sugar pucker on base pair and mispair stability. Biochemistry. 2009;48:11994–2004. 10.1021/bi9014133. 19899814 PMC2814217

[88] Murthy VL, Srinivasan R, Draper DE et al. A complete conformational map for RNA. J Mol Biol. 1999;291:313–27. 10.1006/jmbi.1999.2958. 10438623

[89] Wadley LM, Keating KS, Duarte CM et al. Evaluating and learning from RNA pseudotorsional space: quantitative validation of a reduced representation for RNA structure. J Mol Biol. 2007;372:942–57. 10.1016/j.jmb.2007.06.058. 17707400 PMC2720064

[90] Thibaudeau C, Plavec J, Chattopadhyaya J. Quantitation of the anomeric effect in adenosine and guanosine by comparison of the thermodynamics of the pseudorotational equilibrium of the pentofuranose moiety in N-nucleosides and C-nucleosides. J Am Chem Soc. 1994;116:8033–7. 10.1021/ja00097a010.

[91] Kumar RK, Davis DR. Synthesis and studies on the effect of 2-thiouridine and 4-thiouridine on sugar conformation and RNA duplex stability. Nucleic Acids Res. 1997;25:1272–80. 10.1093/nar/25.6.1272. 9092639 PMC146581

[92] Huang M, Giese TJ, Lee TS et al. Improvement of DNA and RNA sugar pucker profiles from semiempirical quantum methods. J Chem Theory Comput. 2014;10:1538–45. 10.1021/ct401013s. 24803866 PMC3985690

[93] Desaulniers JP, Chui HM, Chow CS. Solution conformations of two naturally occurring RNA nucleosides: 3-methyluridine and 3-methylpseudouridine. Bioorg Med Chem. 2005;13:6777–81. 10.1016/j.bmc.2005.07.061. 16125393

[94] Parisien M, Cruz JA, Westhof E et al. New metrics for comparing and assessing discrepancies between RNA 3D structures and models. RNA. 2009;15:1875–85. 10.1261/rna.1700409. 19710185 PMC2743038

[95] Hopfinger MC, Kirkpatrick CC, Znosko BM. Predictions and analyses of RNA nearest neighbor parameters for modified nucleotides. Nucleic Acids Res. 2020;48:8901–13. 10.1093/nar/gkaa654. 32810273 PMC7498315

[96] Hall KB, McLaughlin LW. Properties of pseudouridine N1 imino protons located in the major groove of an A-form RNA duplex. Nucl Acids Res. 1992;20:1883–9. 10.1093/nar/20.8.1883. 1579489 PMC312302

[97] Scarabino D, Crisari A, Lorenzini S et al. tRNA prefers to kiss. EMBO J. 1999;18:4571–8. 10.1093/emboj/18.16.4571. 10449422 PMC1171531

