# Supplementary material for: Structural analysis of uridine modifications in solved RNA structures
Source: NAR Genom Bioinform. 2026 Jan 10;8(1):lqaf197. doi: 10.1093/nargab/lqaf197 (PMC12789803; doi:10.1093/nargab/lqaf197)
Supplement: lqaf197_Supplemental_File [file lqaf197_supplemental_file.docx]

Supplemental Information for Manuscript Titled:

**Structural Analysis of Uridine Modifications in Solved RNA Structures**

Sebastian J. Arteaga and Brent M. Znosko^*^

Department of Chemistry, Saint Louis University

3501 Laclede Ave., Saint Louis, MO 63103

^*^To whom correspondence should be addressed. Phone: (314) 977-8567. Fax: (314) 977-2521.

Email: [brent.znosko@slu.edu](mailto:brent.znosko@slu.edu)

**Supplemental Table S1. Three-letter abbreviations for modifications in this study and their canonical base equivalents.**

| Three-letter Abbreviation^a^ | Modification | Canonical Base |
| --- | --- | --- |
| PSU | pseudouridine | U |
| 5MU | 5-methyluridine | U/T |
| UR3 | 3-methyluridine | U |
| OMU | O2'-methyluridine | U |
| 4SU | 4-thiouridine | U |
| H2U | 5,6-dihydrouridine | U |
| 2MU | 2',5-dimethyluridine | U |
| SUR | 1-(beta-D-ribofuranosyl)-2-thiouridine | U |
| 1MA | 6-hydro-1-methyladenosine | A |
| 7S3 | 1-methylinosine | A |
| 6IA | N6-isopentenyladenosine | A |
| A2M | O2'-methyladenosine | A |
| OMC | O2'-methylcytidine | C |
| OMG | O2'-methylguanosine | G |

^a^The three-letter abbreviation is the component identifier in the RCSB PDB.

**Supplemental Table S2. Structures excluded from the analysis.**

| PDB ID | Reason for Exclusion |
| --- | --- |
| 3J0D | Missing atoms |
| 4K27 | Missing atoms |
| 4U3M | Unable to be annotated by DSSR |
| 4U3U | Unable to be annotated by DSSR |
| 4U4Q | Unable to be annotated by DSSR |
| 4U4R | Unable to be annotated by DSSR |
| 4U4U | Unable to be annotated by DSSR |
| 4U52 | Unable to be annotated by DSSR |
| 4V88 | Unable to be annotated by DSSR |
| 4V9O | Unable to be annotated by DSSR |
| 4V9P | Unable to be annotated by DSSR |
| 5FL8 | Unable to be annotated by DSSR |
| 5O60 | Unable to clip the secondary structure elements |
| 5O61 | Unable to clip the secondary structure elements |
| 5TBW | Unable to be annotated by DSSR |
| 6I7V | Unable to be annotated by DSSR |
| 6SV4 | Unable to be annotated by DSSR |
| 6Z1P | Unable to be annotated by DSSR |
| 6ZS9 | Missing atoms |
| 7OG4 | Missing atoms |
| 7QVP | Unable to be annotated by DSSR |

**Supplemental Table S3. Secondary structure elements and unique sequences with the sequence-representative structures for modified and unmodified sequences.**

| Modification (3-letter abbrev.)^a^ | Secondary Structure Element | Modified  Sequence (5'-3')^b^ | Freq.^c^ | Modified sequence-representative structure^d^ | Unmodified sequence (5'-3')^b^ | Freq.^c^ | Unmodified sequence-representative structure^d^ |
| --- | --- | --- | --- | --- | --- | --- | --- |
| Pseudouridine (PSU) | Hairpin of 7 | UG**(PSU)(PSU)**CAAAG | 4 | 7UCK_UGPPCAAAG_9_820 | UGUUCAAAG | 2 | 6ZQC_UGUUCAAAG_D3_765 |
|  |  | CGU**(PSU)**CA**(PSU)**AG | 7 | 7O7Y_CGUPCAPAG_B5_4163 | CGUUCAUAG | 232 | 8AGV_CGUUCAUAG_f_2841 |
|  |  | CGU**(PSU)**CAUAG | 2 | 7QIW_CGUPCAUAG_2_2844 |  |  |  |
|  |  | A**(PSU)**GAGAA(OMC)U | 2 | 7QIW_APGAGAAcU_2_1473 | AUGAGAACU | 1 | 6XIQ_AUGAGAACU_1_1463 |
|  |  | GU**(PSU)**CAAAUC | 1 | 7UG7_GUPCAAAUC_Dt_54 | GUUCAAAUC | 6 | 7K53_GUUCAAAUC_6_54 |
|  |  | G(5MU)**(PSU)**CAAAUC | 136 | 8EKB_GtPCAAAUC_1x_53 |  |  |  |
|  |  | G(5MU)**(PSU)**CA(1MA)GUC | 7 | 1F7V_GtPCAaGUC_B_953 | GUUCAAGUC | 4 | 3OV7_GUUCAAGUC_D_13 |
|  |  | G(2MU)**(PSU)**CA(1MA)GUC | 2 | 6SGC_GtPCAaGUC_33_53 |  |  |  |
|  |  | G(5MU)**(PSU)**CAAGUC | 10 | 6V3A_GtPCAAGUC_v_54 |  |  |  |
|  |  | G(5MU)**(PSU)**CG(1MA)AUC | 1 | 1H3E_GtPCGaAUC_B_53 | GUUCGAAUC | 5 | 3CUN_GUUCGAAUC_D_170 |
|  |  | G(5MU)**(PSU)**CGAAUC | 14 | 7N1P_GtPCGAAUC_Pt_53 |  |  |  |
|  |  | GU**(PSU)**CGAAUC | 1 | 7O1A_GUPCGAAUC_B8_53 |  |  |  |
|  |  | G(5MU)**(PSU)**CG(1MA)CUC | 1 | 2DLC_GtPCGaCUC_Y_553 | GUUCGACUC | 2 | 3IYQ_GUUCGACUC_A_327 |
|  |  | G(5MU)**(PSU)**CGACUC | 4 | 6GSJ_GtPCGACUC_1K_53 |  |  |  |
|  |  | G(5MU)**(PSU)**CGAGUC | 6 | 3KFU_GtPCGAGUC_L_53 | GUUCGAGUC | 3 | 4X4P_GUUCGAGUC_B_13 |
|  |  | G(SUR)**(PSU)**(OMC)(7S3)(1MA)GUC | 2 | 7VNV_GuPcgaGUC_B_53 |  |  |  |
|  |  | GU**(PSU)**(OMC)(7S3)(1MA)GUC | 2 | 7VNW_GUPcgaGUC_B_53 |  |  |  |
|  |  | G(5MU)**(PSU)**CGAUUC | 141 | 4WPO_GtPCGAUUC_BW_53 | GUUCGAUUC | 15 | 4V9D_GUUCGAUUC_AV_54 |
|  |  | GU**(PSU)**CGAUUC | 3 | 7UG7_GUPCGAUUC_Pt_53 |  |  |  |
|  |  | A**(PSU)**UGAAAAU | 1 | 4WSD_APUGAAAAU_3L_31 | AUUGAAAAU | 6 | 6HTQ_AUUGAAAAU_w_32 |
|  |  | A**(PSU)**UGAA(6IA)AU | 1 | 5IQR_APUGAAaAU_6_31 |  |  |  |
|  |  | CUUGAGG**(PSU)**G | 1 | 1N32_CUUGAGGPG_Y_31 | CUUGAGGUG | 5 | 7NSO_CUUGAGGUG_8_33 |
|  |  | C**(PSU)**UGUC(1MG)CG | 8 | 1ASZ_CPUGUCgCG_S_631 | CUUGUCGCG | 4 | 3J16_CUUGUCGCG_L_32 |
|  |  |  |  |  |  |  |  |
|  | Hairpin of 6 | A(A2M)GAAA**(PSU)**U | 3 | 7OW7_AaGAAAPU_A_3723 | AAGAAAUU | 3 | 5JCS_AAGAAAUU_x_2220 |
|  |  | (A2M)AGAAA**(PSU)**U | 3 | 7UCK_aAGAAAPU_5_3723 |  |  |  |
|  |  | GAGCGA**(PSU)**U | 1 | 7QIZ_GAGCGAPU_S2_1298 | GAGCGAUU | 1 | 7SYI_GAGCGAUU_2_1353 |
|  |  | G**(PSU)**GAAAGC | 2 | 7QIW_GPGAAAGC_2_2747 | GUGAAAGC | 3 | 6HCM_GUGAAAGC_52_4321 |
|  |  | G**(PSU)**UAAUUC | 9 | 7O7Z_GPUAAUUC_A2_1367 | GUUAAUUC | 116 | 7SYX_GUUAAUUC_2_1367 |
|  | Hairpin of 5 | UUG**(PSU)**UCA | 3 | 7OYD_UUGPUCA_5_4501 | UUGUUCA | 41 | 7A5J_UUGUUCA_A_3039 |
|  |  | U(OMU)(OMG)**(PSU)**UCA | 10 | 7ZJW_UugPUCA_L5_4243 |  |  |  |
|  | Hairpin of 4 | U**(PSU)**ACAA | 2 | 7QIZ_UPACAA_2_67 | UUACAA | 12 | 7MT2_UUACAA_A_2595 |
|  |  | CC**(PSU)**CAG | 13 | 7OYD_CCPCAG_5_1679 | CCUCAG | 228 | 6R5Q_CCUCAG_5_1676 |
| 5-methyluridine (5MU) | Hairpin of 7 | G**(5MU)**UCGAUUC | 1 | 4WSD_GtUCGAUUC_1L_53 | GUUCGAUUC | 15 | 4V9D_GUUCGAUUC_AV_54 |
|  |  | G**(5MU)**(PSU)CGAUUC | 141 | 4WPO_GtPCGAUUC_BW_53 |  |  |  |
|  |  | G**(5MU)**(PSU)CGAGUC | 6 | 3KFU_GtPCGAGUC_L_53 | GUUCGAGUC | 3 | 4X4P_GUUCGAGUC_B_13 |
|  |  | G**(5MU)**(PSU)CG(1MA)CUC | 1 | 2DLC_GtPCGaCUC_Y_553 | GUUCGACUC | 2 | 3IYQ_GUUCGACUC_A_327 |
|  |  | G**(5MU)**(PSU)CGACUC | 4 | 6GSJ_GtPCGACUC_1K_53 |  |  |  |
|  |  | G**(5MU)**(PSU)CGAAUC | 14 | 7N1P_GtPCGAAUC_Pt_53 | GUUCGAAUC | 5 | 3CUN_GUUCGAAUC_D_170 |
|  |  | G**(5MU)**(PSU)CG(1MA)AUC | 1 | 1H3E_GtPCGaAUC_B_53 |  |  |  |
|  |  | G**(5MU)**(PSU)CA(1MA)GUC | 7 | 1F7V_GtPCAaGUC_B_953 | GUUCAAGUC | 4 | 3OV7_GUUCAAGUC_D_13 |
|  |  | G**(5MU)**(PSU)CAAGUC | 10 | 6V3A_GtPCAAGUC_v_54 |  |  |  |
|  |  | G**(5MU)(5MU)**CAAG**(5MU)**C | 1 | 7OT5_GttCAAGtC_z_62 |  |  |  |
|  |  | G**(5MU)**UCAAAUC | 1 | 4V51_GtUCAAAUC_AV_53 | GUUCAAAUC | 6 | 7K53_GUUCAAAUC_6_54 |
|  |  | G**(5MU)**(PSU)CAAAUC | 136 | 8EKB_GtPCAAAUC_1x_53 |  |  |  |
|  | Hairpin of 6 | **(5MU)**GCAAGGG | 1 | 6SKG_tGCAAGGG_BA_2427 | UGCAAGGG | 4 | 6SKF_UGCAAGGG_BA_2428 |
|  | Hairpin of 4 | **(5MU)**GUAAG | 1 | 6SKF_tGUAAG_BA_1085 | UGUAAG | 13 | 4V6U_UGUAAG_B1_1093 |
| 3-methyluridine (UR3) | Hairpin of 5 | GG**(UR3)**AAGC | 4 | 7F5S_GGuAAGC_L5_1864 | GGUAAGC | 282 | 6Y6X_GGUAAGC_L5_1865 |
| O2'-methyluridine (OMU) | Hairpin of 7 | **(OMU)**UCAG**(OMU)**GUG | 1 | 7MPJ_uUCAGuGUG_A1_2724 | UUCAGUGUG | 98 | 7ZW0_UUCAGUGUG_LA_2725 |
|  |  | UUCAG**(OMU)**ACG | 10 | 7O7Z_UUCAGuACG_B5_4047 | UUCAGUACG | 15 | 6OM7_UUCAGUACG_t_4264 |
|  |  | **(OMU)**CCAGUA(OMC)G | 2 | 3JCS_uCCAGUAcG_2_1153 | UCCAGUACG | 5 | 5T5H_UCCAGUACG_B_1285 |
|  |  | GG**(OMU)**GUUCAC | 2 | 6SKG_GGuGUUCAC_BA_2654 | GGUGUUCAC | 2 | 4V4N_GGUGUUCAC_A1_2666 |
|  |  | GG**(OMU)**(OMG)UUCAC | 1 | 6SKF_GGugUUCAC_BA_2654 |  |  |  |
|  |  | GGCA**(OMU)**CGGC | 3 | 6TH6_GGCAuCGGC_BA_449 | GGCAUCGGC | 2 | 4V4N_GGCAUCGGC_'A1'450 |
|  |  | C(OMC)U**(OMU)**ACAAG | 4 | 7VNV_CcUuACAAG_B_31 | CCUUACAAG | 5 | 7EQJ_CCUUACAAG_B_32 |
|  | Hairpin of 5 | U**(OMU)**(OMG)(PSU)UCA | 10 | 7ZJW_UugPUCA_L5_4243 | UUGUUCA | 386 | 7A5J_UUGUUCA_A_3039 |
|  |  | U**(OMU)**(OMG)UUCA | 8 | 7OF7_UugUUCA_A_3038 |  |  |  |
|  |  | **(OMU)**GAGAAG | 3 | 6SKG_uGAGAAG_BA_1776 | UGAGAAG | 2 | 4V6U_UGAGAAG_B1_1788 |
|  |  | C**(OMU)**GUUCG | 130 | 7RQ8_CuGUUCG_1A_2551 | CUGUUCG | 41 | 7ZOD_CUGUUCG_b_2552 |
|  | Hairpin of 4 | GGUAA**(OMU)** | 1 | 6AZ3_GGUAAu_3_5 | GGUAAU | 2 | 5T2A_GGUAAU_E_6 |
|  |  | CC**(OMU)**CAG | 1 | 5T5H_CCuCAG_A_1125 | CCUCAG | 230 | 6R5Q_CCUCAG_5_1676 |
|  |  | **(OMU)**GAAAG | 1 | 6SKF_uGAAAG_BA_768 | UGAAAG | 521 | 6LQM_UGAAAG_2_4322 |
| 4-thiouridine (4SU) | Hairpin of 7 | AGCCCA**(4SU)**AU | 2 | 6SKF_AGCCCAuAU_BA_2575 | AGCCCAUAU | 19 | 4V4N_AGCCCAUAU_A1_2587 |
|  | Hairpin of 6 | **(4SU)**GGAGCAG | 3 | 5WDT_uGGAGCAG_w_8 | UGGAGCAG | 3 | 7MT2_UGGAGCAG_A_2767 |
| 5,6-dihydrouridine (H2U) | Hairpin of 7 | CAG**(H2U)**GGAAG | 2 | 6TB3_CAGuGGAAG_n_13 | CAGUGGAAG | 1697 | 7NRD_CAGUGGAAG_Sn_14 |
|  | Hairpin of 4 | CG**(H2U)**GAG | 1 | 3JCS_CGuGAG_2_1402 | CGUGAG | 12 | 7QIW_CGUGAG_2_2968 |

^a^The three-letter abbreviation used in the RCSB PDB database.

^b^Underlined residues make up the unpaired nucleotides in the hairpin. The first and last residues make up the first closing base pair of the hairpin. For the modified sequences, modifications are in parentheses, and the bolded residues highlight the uridine modification of interest in the SSE. All modifications are denoted using their RCSB PDB three-letter abbreviation. These modification descriptions can be found in Supplemental Table S1.

^b^Frequency of occurrence for unique sequences.

^d^Sequence-representative structure identifiers are formatted as follows, with components separated by underscores: four-letter PDB ID, SSE sequence including the closing base pair (lowercase letters indicate modification sites; an uppercase “P” denotes a pseudouridine modification), chain ID from the original *.cif file, and the residue index of the first closing base pair on the 5’ side in the original *.cif file. Some modified SSEs share an unmodified sequence-representative structure. For SSE comparisons, all modified residues, except for the six uridine modifications of interest, were mapped to their canonical nucleotides, as detailed in Supplemental Table S1. Canonical bases were also used to identify modified residues during RNA CoSSMos database searches.


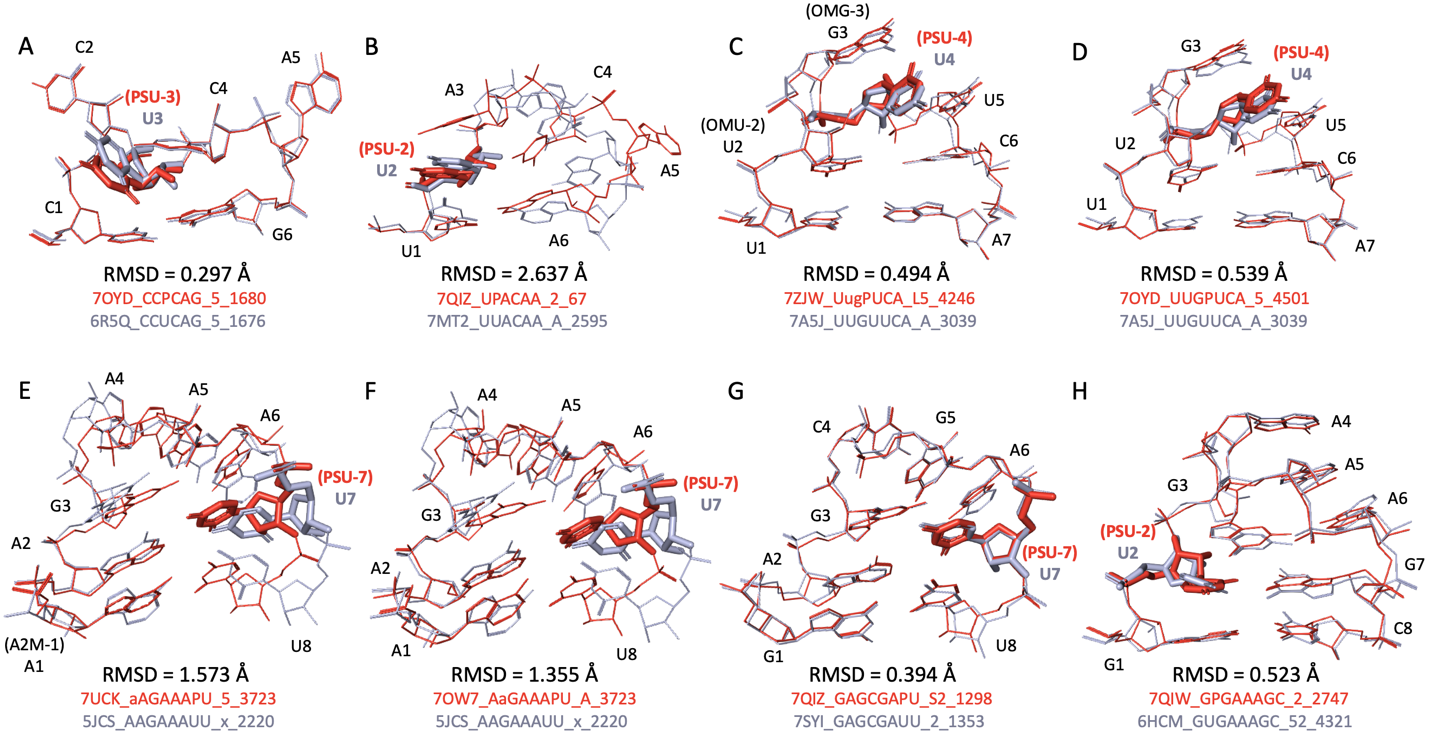

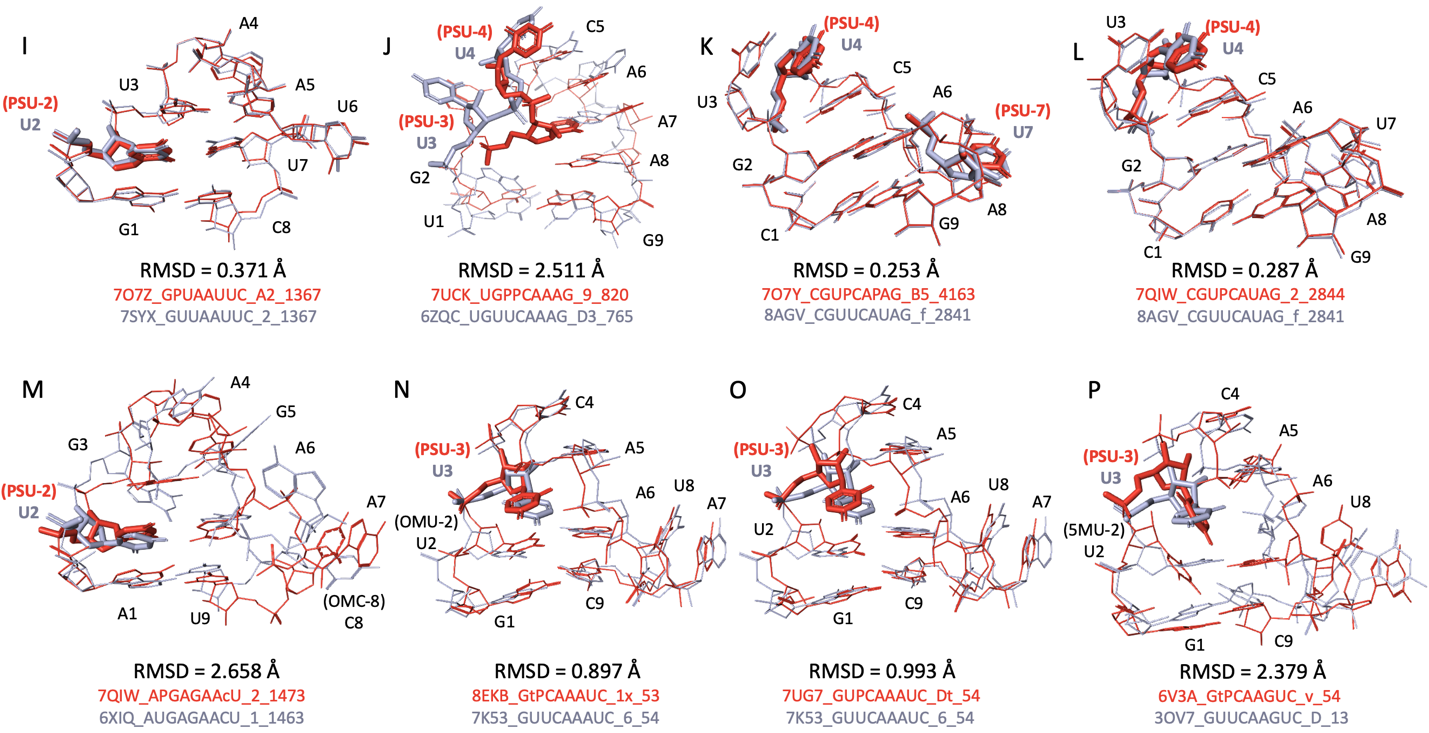

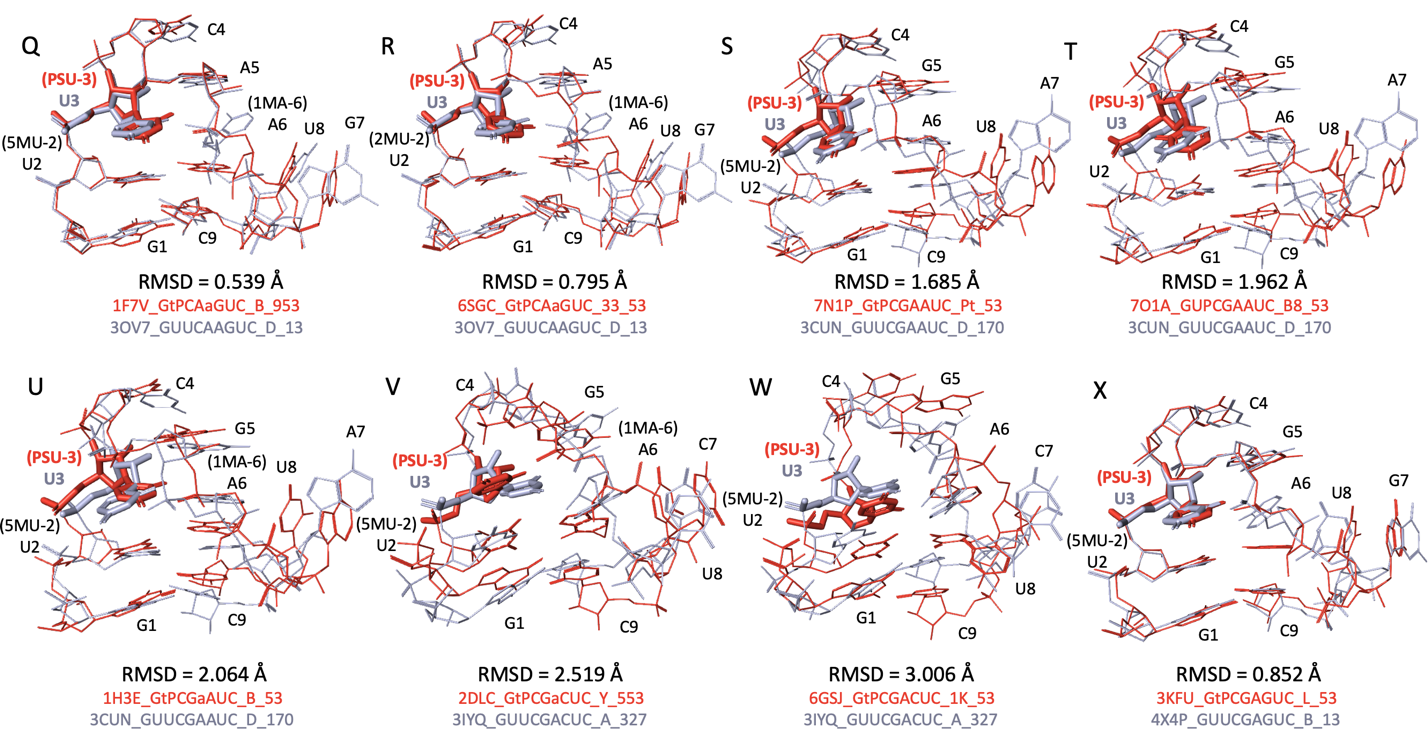


**Supplemental Figure S1. See next page for caption.**


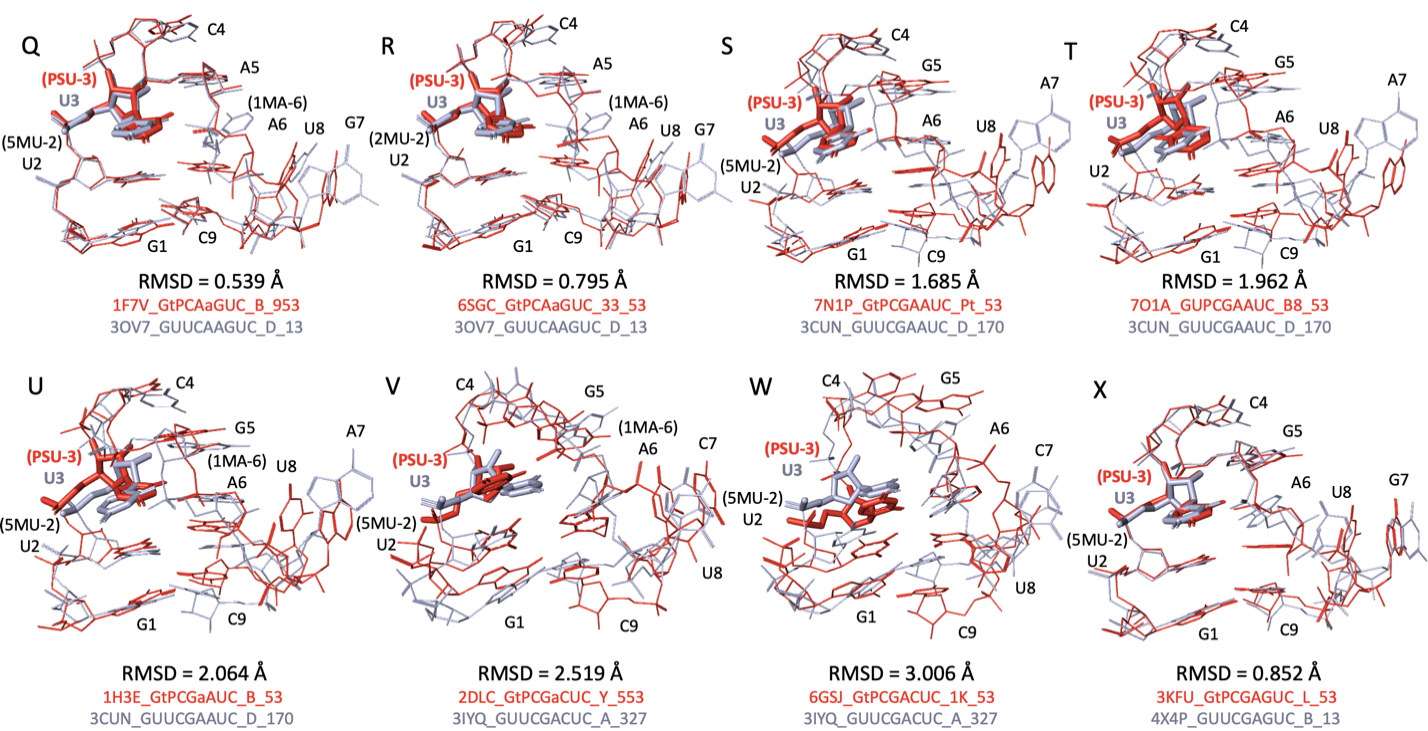


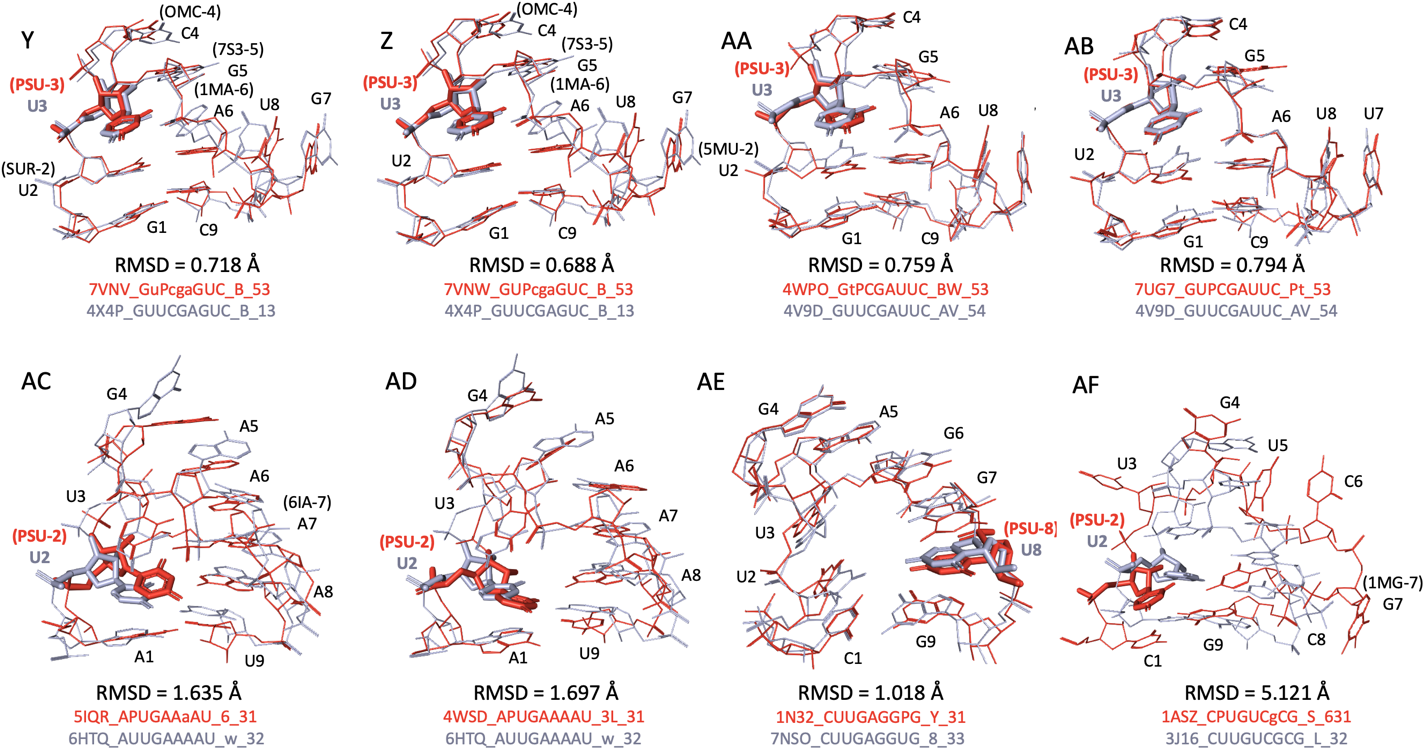


**Supplemental Figure S1. Structural comparison of sequence-representative structures containing pseudouridine and uridine.** Tetraloop structures resembling (**A**) the YGAR cluster family (64) and (**B**) unclustered structures. (**C and D**) Pentaloop structures from the UGUUC cluster family (65). (**E-I**) Hexaloop structures, and (**J-AF**) Heptaloop structures. In each panel, the structure containing pseudouridine (red) is superimposed onto the corresponding unmodified structures (light blue). Residue numbering begins at the closing base pair on the 5’ side. Modified nucleotides are annotated with their three-letter PDB codes in parentheses; the corresponding canonical (unmodified) residues are shown below the modification site. The residue of interest is bolded and rendered as sticks, while all other residues are shown as thin wire representations. Structural similarity between the modified and unmodified forms is quantified using all-atom RMSD. Below each RMSD value, identifying information is provided with components separated by underscores: four-letter PDB ID, SSE sequence including the closing base pair (lowercase letters indicate modification sites; an uppercase “P” denotes a pseudouridine modification), chain ID from the original *.cif file, and the residue index of the first closing base pair on the 5’ side in the original *.cif file.


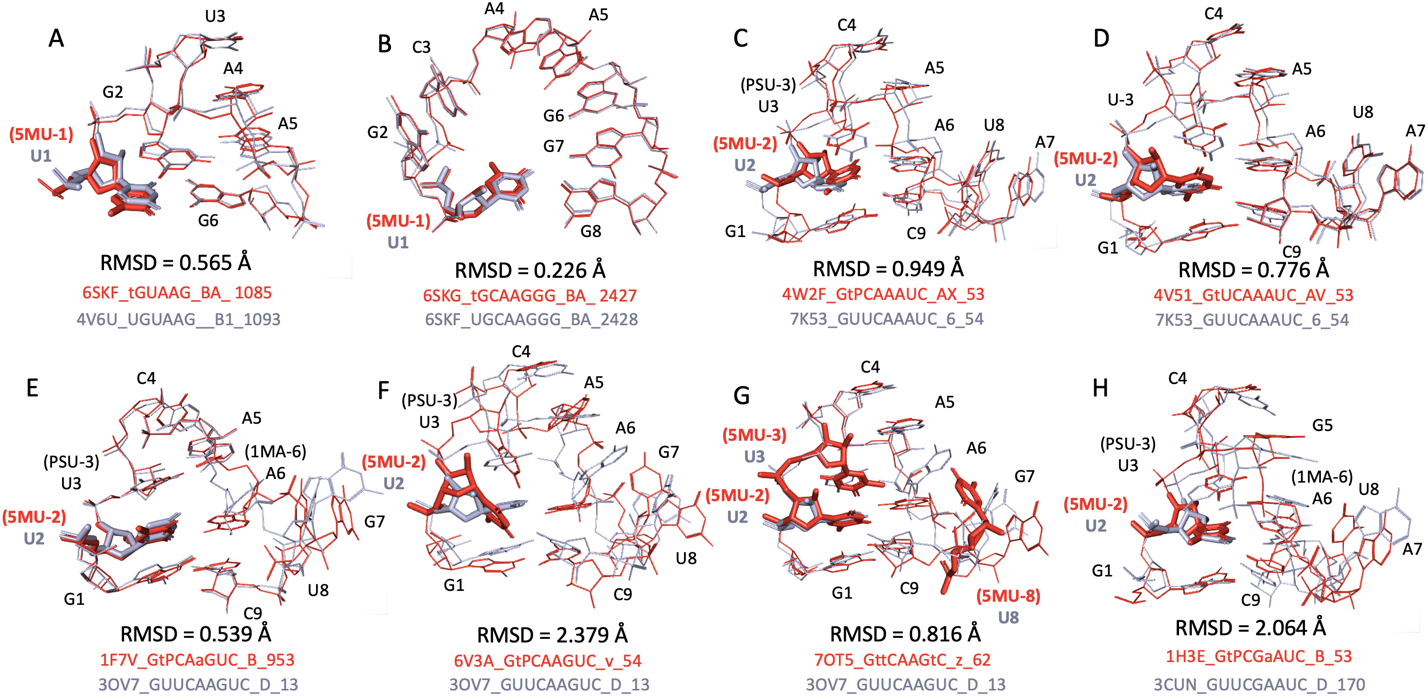

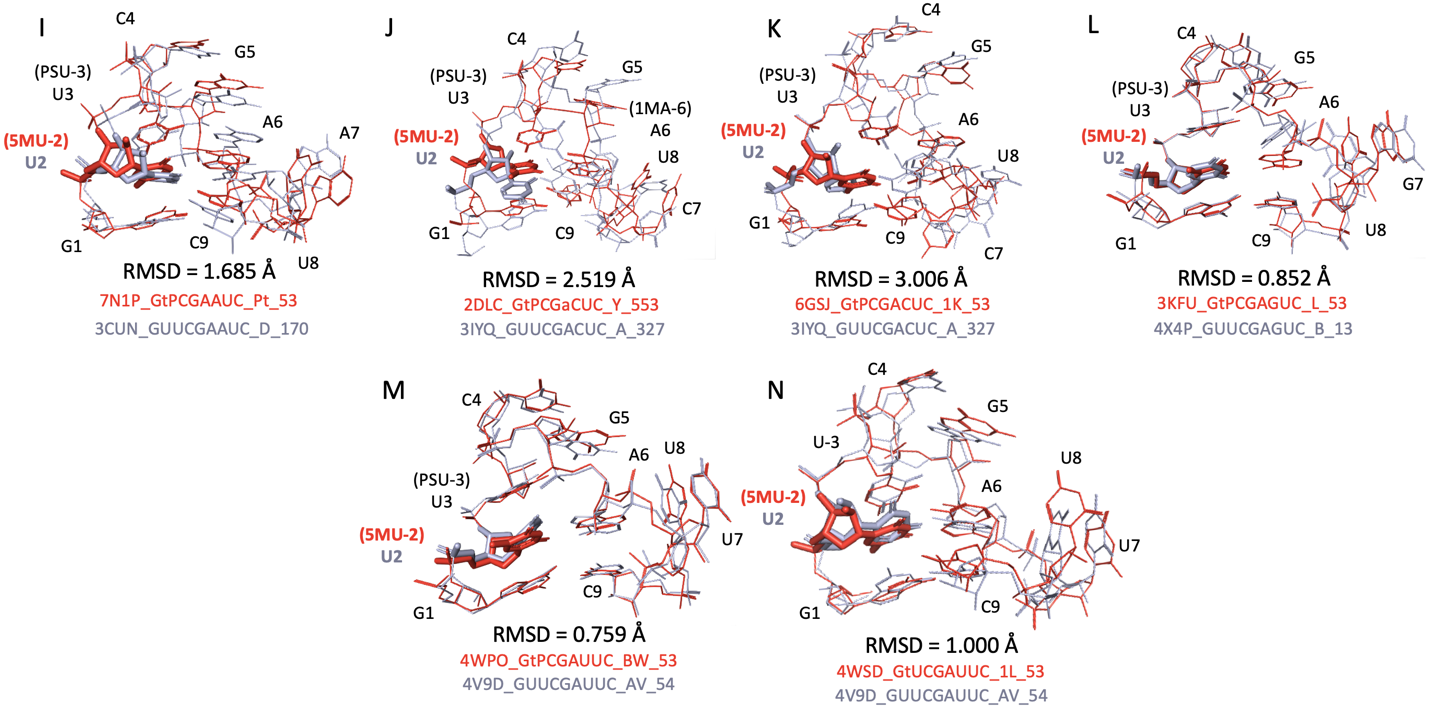


**Supplemental Figure S2. Structural comparison of sequence-representative structures containing 5-methyluridine and uridine.** (**A**) A tetraloop from the GNRA family (64) is shown with a U-G closing base pair. (**B**) A hexaloop with a U-G closing base pair. (**C-N**) Additional heptaloops, all containing G-C closing base pairs. In each panel, structures containing 5-methyluridine (red) are superimposed onto corresponding unmodified uridine-containing structures (light blue). Residue numbering within each secondary structure element begins at the closing base pair on the 5’ side. Modified nucleotides are annotated with their three-letter PDB codes in parentheses; the corresponding canonical (unmodified) residues are shown below the modification site. The modified residue is shown in bold and rendered as sticks; all other nucleotides are shown as wires. Global structural similarity was quantified using all-atom RMSD. Below each RMSD value, the corresponding structure identifier is provided with components separated by underscores: four-letter PDB ID, SSE sequence including the closing base pair (lowercase letters indicate modification sites; a lowercase “t” denotes a thymine base on a ribose sugar; an uppercase “P” denotes a pseudouridine modification), chain ID from the original *.cif file, and the residue index of the first closing base pair on the 5’ side in the original *.cif file.


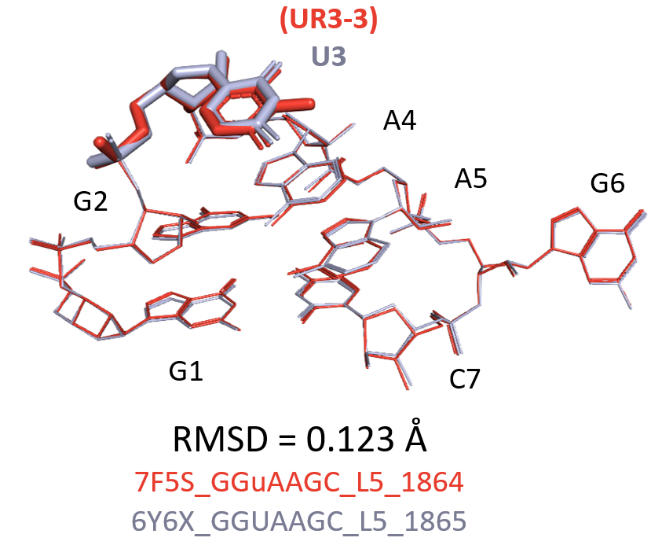


**Supplemental Figure S3. Structural comparison of sequence-representative pentaloops containing 3-methyluridine and uridine**. A representative pentaloop structure with a G-C closing base pair is shown, comparing the modified structure containing 3-methyluridine (red) to the unmodified structure with uridine (light blue). Structures are superimposed to highlight conformational differences. Residue numbering begins at the 5’ closing base pair. Modified nucleotides are annotated with their three-letter PDB codes in parentheses; the corresponding canonical (unmodified) residues are shown below the modification site. The residue of interest is shown in bold and rendered as sticks; all other residues are shown as wires and not bolded. All-atom RMSD values quantify global structural similarity between the two structures. Below each RMSD value is the identifying information for the compared structures, with components separated by underscores: four-letter PDB ID, SSE sequence including the closing base pair (lowercase letters indicate modification sites), chain ID from the original *.cif file, and the residue index of the first closing base pair on the 5’ side in the original *.cif file.

**
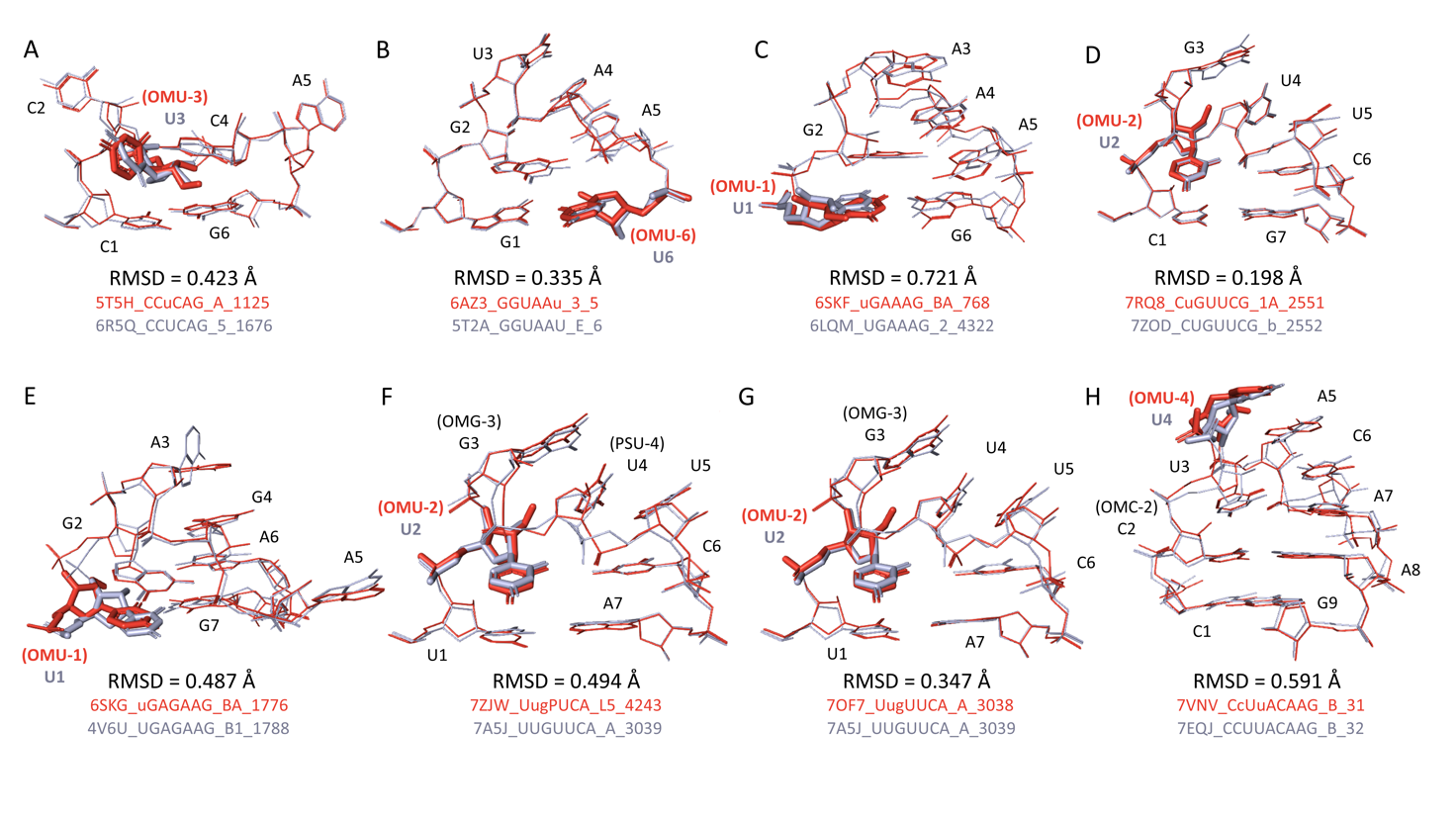
**
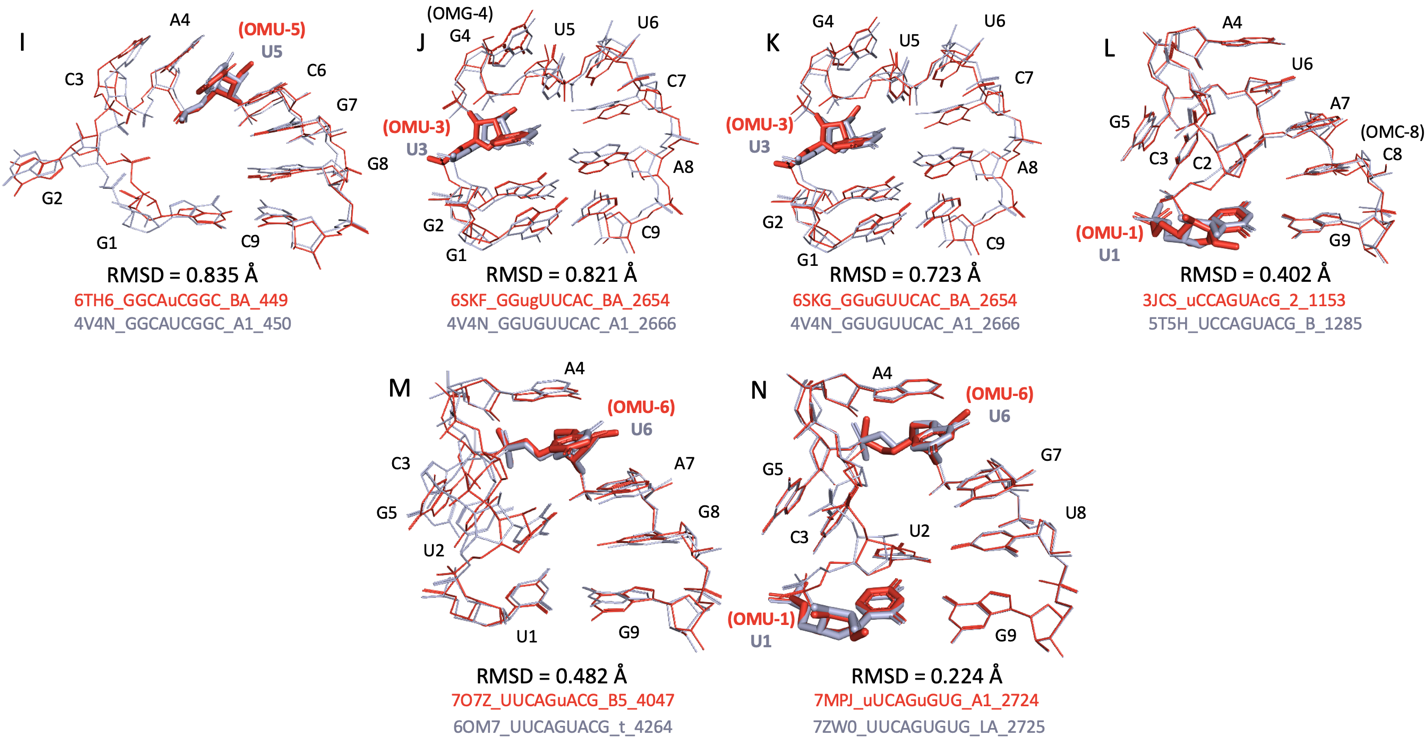
**Supplemental Figure S4. Structural comparison of sequence-representative structures containing O2’-methyluridine and uridine.** (**A-C**) Tetraloop structures resembling members of the (**A**) YGAR and (**B** and **C**) GNRA cluster families (64). (**D-G**) Pentaloop structures from the (**D, F,** and **G**) UGUUC cluster family and one (**E**) similar to the UAAMK family (65). (**H-N**) Heptaloop structures. In each panel, structures containing O2’-methyluridine (red) are superimposed onto their unmodified uridine counterparts (light blue). Residue numbering within each secondary structure begins at the closing base pair on the 5’ side. Modified nucleotides are annotated with their three-letter PDB codes in parentheses; the corresponding canonical (unmodified) residues are shown below the modification site. The residue of interest is bolded and displayed as sticks, while all other residues are shown as wires. Global structural similarity between the modified and unmodified structures was assessed using all-atom RMSD. Below each RMSD value is the identifier for the corresponding structure with components separated by underscores: four-letter PDB ID, SSE sequence including the closing base pair (lowercase letters indicate modification sites; an uppercase “P” denotes a pseudouridine modification), chain ID from the original *.cif file, and the residue index of the first closing base pair on the 5’ side in the original *.cif file.


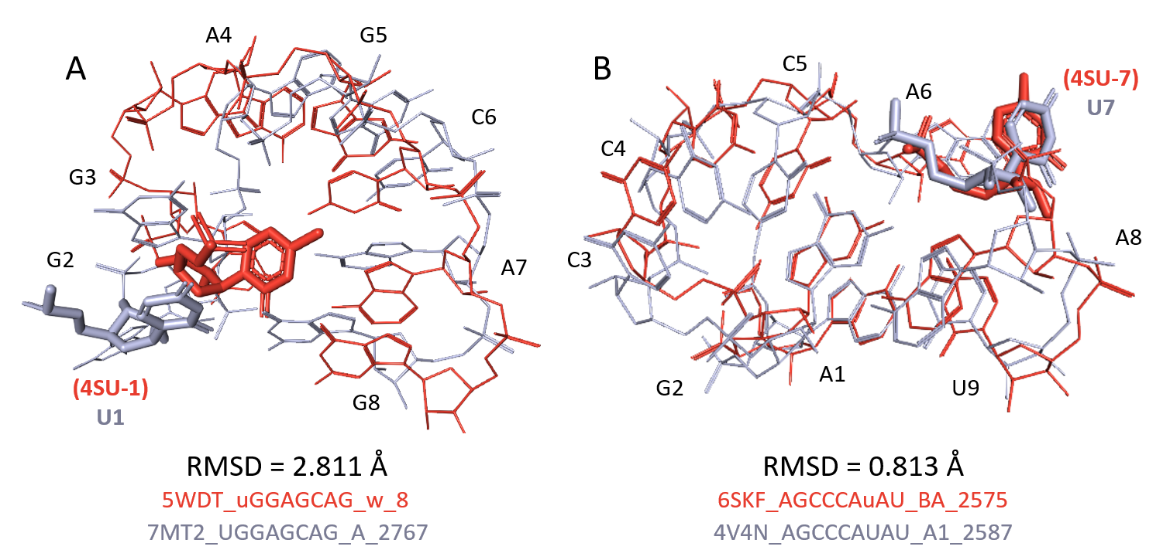


**Supplemental Figure S5. Structural comparison of sequence-representative hexaloop and heptaloop containing 4-thiouridine and uridine.** (**A**) A hexaloop structure is shown with a 4-thiouridine-G (4SU-G) closing pair, compared to an unmodified U-G pair. The modified structure (red) is superimposed on the unmodified counterpart (light blue). (**B**) A heptaloop structure with an A-U closing base pair is shown using the same color scheme. Residue numbering begins at the 5’ closing base pair. Modified nucleotides are annotated with their three-letter PDB codes in parentheses; the corresponding canonical (unmodified) residues are shown below the modification site. The residue of interest is shown in bold and rendered as sticks; all other residues are depicted as wires and are not bolded. All-atom RMSD values quantify the global structural similarity between each pair of structures. Below each RMSD value is the identifying information for the compared structures formatted with components separated by underscores: four-letter PDB ID, SSE sequence including the closing base pair (lowercase letters indicate modification sites), chain ID from the original *.cif file, and the residue index of the first closing base pair on the 5’ side in the original *.cif file.


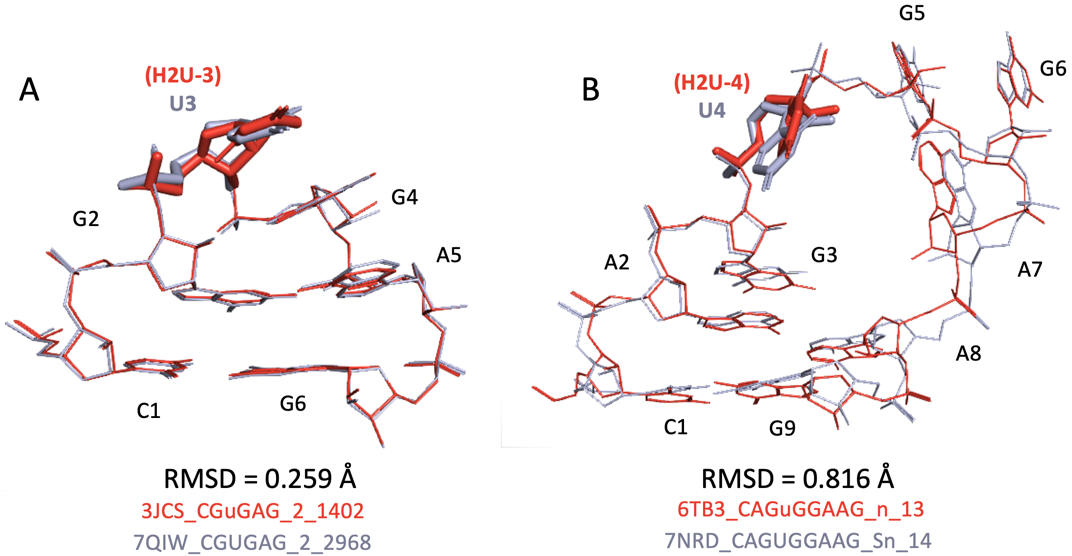


**Supplemental Figure S6. Structural comparison of sequence-representative structures containing 5,6-dihydrouridine and uridine.** (**A**) A tetraloop structure from the GNRA cluster family (64) is shown with a C-G closing base pair. The modified structure containing 5,6-dihydrouridine (red) is superimposed onto the unmodified uridine-containing structure (light blue). (**B**) A heptaloop structure with a C-G closing base pair is shown, with residue numbering beginning at the 5’-side of the closing base pair. Modified nucleotides are annotated with their three-letter PDB codes in parentheses; the corresponding canonical (unmodified) residues are shown below the modification site. The residue of interest is bolded and shown as sticks; all other residues are shown as wires and not bolded. Global structural similarity was assessed using an all-atom RMSD calculation. The RMSD value is shown above the identifying information for each structure, with components separated by underscores: four-letter PDB ID, SSE sequence including the closing base pair (lowercase letters indicate modification sites), chain ID from the original *.cif file, and the residue index of the first closing base pair on the 5’ side in the original *.cif file.
